# Recent progress on flat plate solar collectors equipped with nanofluid and turbulator: state of the art

**DOI:** 10.1007/s11356-023-29815-9

**Published:** 2023-10-04

**Authors:** Mohammad Zaboli, Seyfolah Saedodin, Seyed Soheil Mousavi Ajarostaghi, Nader Karimi

**Affiliations:** 1 Faculty of Mechanical Engineering, Semnan University, Semnan, Iran; 2 Mechanical Engineering Department, Université de Sherbrooke, Sherbrooke, QC J1K 2R1 Canada; 3 School of Engineering and Material Science, Queen Mary University of London, London, E1 4NS UK

**Keywords:** Flat plate solar collectors (FPSCs), Nanofluid, Turbulator, Heat transfer enhancement, Passive methods

## Abstract

This paper reviews the impacts of employing inserts, nanofluids, and their combinations on the thermal performance of flat plate solar collectors. The present work outlines the new studies on this specific kind of solar collector. In particular, the influential factors upon operation of flat plate solar collectors with nanofluids are investigated. These include the type of nanoparticle, kind of base fluid, volume fraction of nanoparticles, and thermal efficiency. According to the reports, most of the employed nanofluids in the flat plate solar collectors include Al_2_O_3_, CuO, and TiO_2_. Moreover, 62.34%, 16.88%, and 11.26% of the utilized nanofluids have volume fractions between 0 and 0.5%, 0.5 and 1%, and 1 and 2%, respectively. The twisted tape is the most widely employed of various inserts, with a share of about one-third. Furthermore, the highest achieved flat plate solar collectors’ thermal efficiency with turbulator is about 86.5%. The review is closed with a discussion about the recent analyses on the simultaneous use of nanofluids and various inserts in flat plate solar collectors. According to the review of works containing nanofluid and turbulator, it has been determined that the maximum efficiency of about 84.85% can be obtained from a flat plate solar collector. It has also been observed that very few works have been done on the combination of two methods of employing nanofluid and turbulator in the flat plate solar collector, and more detailed work can still be done, using more diverse nanofluids (both single and hybrid types) and turbulators with more efficient geometries.

## Introduction

The worldwide need for energy goes up constantly. Increasing the energy demand has got up the consumption of fossil fuels. Due to the supply of energy resources, environmental considerations, the use of petrochemicals and the increasing price of fossil fuels, and technological progress, developing and developed countries have a particular view on renewable energy. Converting a natural phenomenon into a proper type of energy is defined as renewable energy technologies. One of the factors that can be stated for using renewable energy instead of fossil energy is the issue of reducing the emission of toxic gases and keeping the environment safe (Georgeson et al. 2017). In general, renewable energies such as solar, wind, and geothermal energy do not pollute the environment and are compatible with nature. Research related to renewable energy sources has gone up widely in recent years.

Sunlight is the source of most of the energy on earth. As a natural nuclear reactor, the sun releases photon energy, which travels a distance of 150 million kilometers from the sun to the earth in approximately 8.5 min. In the past, solar energy was the only energy used by humans (Anderson 1977) (Kreith and Kreider 1978). Exploiting energy from the sun delivers a desirable contamination-free solution for supplying heating systems; thus, solar energy can be expressed as the most prevalent source. It should also be noted that the sun’s energy can be used directly and indirectly to produce different types of energy, such as heat and electricity. The primary challenges of utilizing solar energy arise from its widespread availability and fluctuating nature. Solar energy is categorized into power plant and non-power plant indicators based on its specific application. Solar dryers, solar water heaters, and solar water desalination are among the most critical non-power plant applications (Alawaji 2001). Additionally, production of hot water stands out as a crucial application of solar energy due to its favorable economics. Rewarding the significance of solar energy, increasing the efficiency of this energy has obtained growing consideration. One of the most challenging issues in enhancing the thermal efficiency of solar collectors is trying to absorb as much solar radiation energy as possible. For this purpose, a solar energy tracker and a suitable designer are needed (Motahhir et al. 2019). Generally, solar collectors are one of the particular heat transfer devices that transfer the sun’s radiant energy to the internal energy of the carrier environment. The solar collector is responsible for absorbing the energy of the sun’s radiation and converting it into the required heat of the fluid. The two basic types of solar collectors are decentralized or fixed and centralized. In decentralized collectors, the process of absorbing solar energy is done by a single surface. While in the concentrated type, direct solar radiation is received by reflective concave surfaces and concentrated on a smaller surface. Recently, a lot of endeavors have been made by researchers to go up the collector’s efficiency.

## Types of solar collectors

All solar collectors are categorized into three main groups according to the maximum temperature produced (fluid outlet temperature). This classification includes solar collectors with low temperatures (temperatures less than 100 ℃), medium temperatures (temperatures between 100 and 300 ℃), and high temperatures (temperatures more than 300 ℃) (Kalogirou 2004). The high-temperature collector is utilized for power plant applications; the medium-temperature collector is employed for food industries, hospitals, and office applications; and the low-temperature collector is implemented as a solar water heater.

### Flat plate solar collectors (FPSCs)

Flat plate solar collectors are one of the most common and widely used solar collector models. They are commonly utilized in low-temperature heating applications owing to their simple design (it consists of a flat, rectangular box-like structure with a transparent cover, typically made of glass or plastic, that allows sunlight to pass through. Inside the collector, there is an absorber plate, usually made of metal, which is painted black to maximize its ability to absorb solar radiation), effortless installation, and lower cost than other models (Mustafa et al. 2021). For the reasons mentioned, according to reports spanning from 2011 to 2021, the utilization of flat plate solar collectors witnessed a 15% increase, constituting 35% of all solar collectors employed during that period. The importance of using this type of collector is determined when this number was reported to be nearly 72% for Europe (Weiss and Mauthner 2010).

**Fig. 1:**
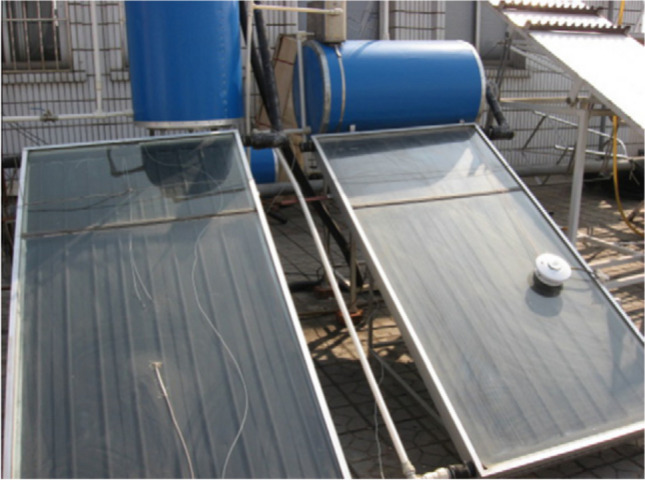
A view of the flat plate collector (Tang et al. 2010)

Flat plate collectors are usually placed in a fixed position and do not need to follow the sun. Also, their placement direction is usually directly along the equator, towards the north in the southern hemisphere, and towards the south in the northern hemisphere. The collector curvature angle is equal to the latitude position with a deviation angle of more or less than 10 to 15°, which depends on its application (Kalogirou 2004). For instance, a picture of the flat plate collector is demonstrated in Fig. 1.

### Parabolic tube solar collectors (PTSCs)

**Fig.2:**
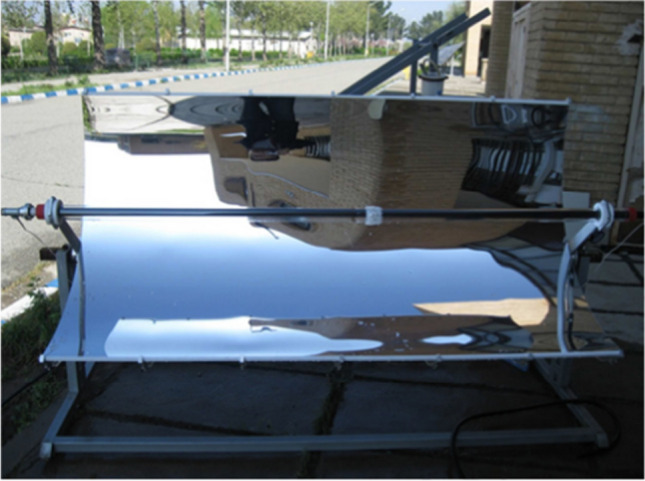
A view of the parabolic solar collector (Jamal-Abad et al. 2017)

A parabolic solar collector, also known as a parabolic trough collector, is a type of solar thermal technology used to harness solar energy for various applications. The parabolic collector is one of the most widely used types of collectors; installed collector areas (end of March 2023) are reported to be 670,000 m^2^ (Weiss and Mauthner 2010). The parabolic shape of the reflector allows the concentration of incoming sunlight into a receiver tube positioned along the focal line of the trough. As the sunlight is reflected off the parabolic surface, it converges towards the receiver tube, maximizing the amount of solar energy captured (Nazir et al. 2021). Synthetic oil was used as a heat transfer fluid in the first parabolic collector solar power plants (Pal and Kumar 2021). The most critical applications of the linear parabolic collector can be mentioned in heating and cooling loops, drying processes, power generation plants, and desalination (Nawsud et al. 2022). For instance, a picture of the parabolic solar collector is displayed in Fig. 2.

### Linear Fresnel solar collector (LFSCs)

**Fig. 3:**
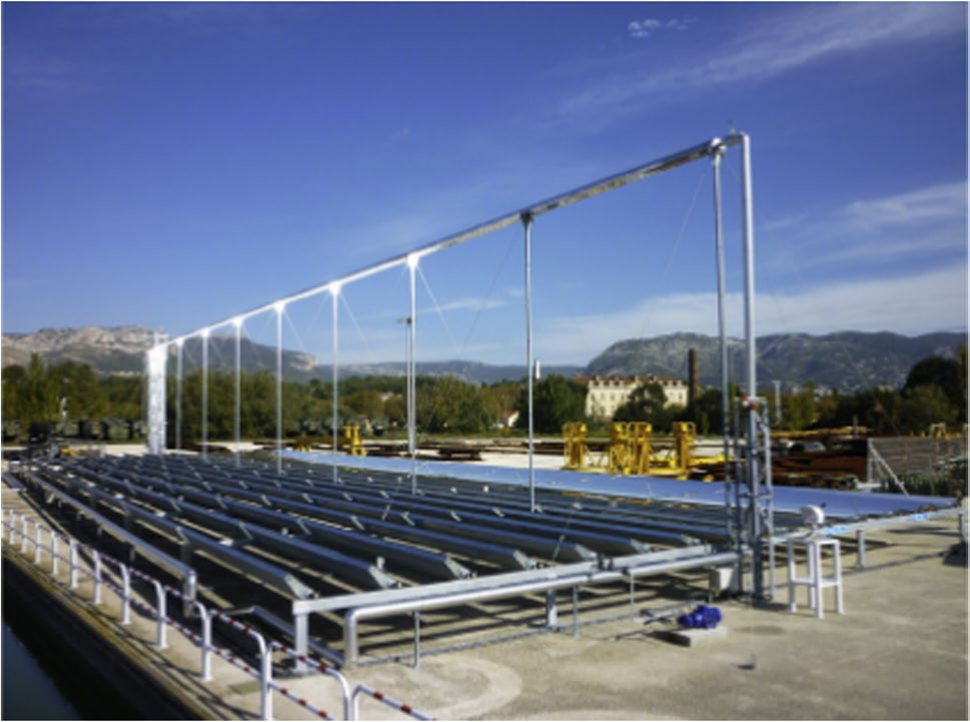
A view of the Fresnel solar collector (Beltagy et al. 2017)

A linear Fresnel solar collector is a type of solar thermal technology that utilizes a series of flat mirrors with a small width and a large length and with a fixed receiver to concentrate sunlight onto an absorber tube. It is named after the French physicist Augustin-Jean Fresnel, who invented the Fresnel lens. Linear Fresnel solar collectors use a curved mirror to concentrate sunlight onto a single focal line, Linear Fresnel collectors focus sunlight along multiple lines. This design allows for a wider absorption area and reduces the need for complex tracking systems. Linear Fresnel collectors are often used in large-scale solar thermal power plants. It can be said that the length of these collectors is more than 100 m. One advantage of linear Fresnel collectors is their modular design, allowing for easier installation and maintenance than other concentrated solar power technologies. The linear arrangement of mirrors simplifies their manufacturing and assembly. Also, the core disadvantage of this type of collector can be considered low optical performance (Rungasamy et al. 2021). It should be noted that the installed area of this type of collector reaches 24,000 m^2^ (Weiss and Mauthner 2010). For instance, a picture of the Fresnel solar collector is displayed in Fig. 3.

### Evacuated tube solar collectors (ETSCs)

**Fig. 4:**
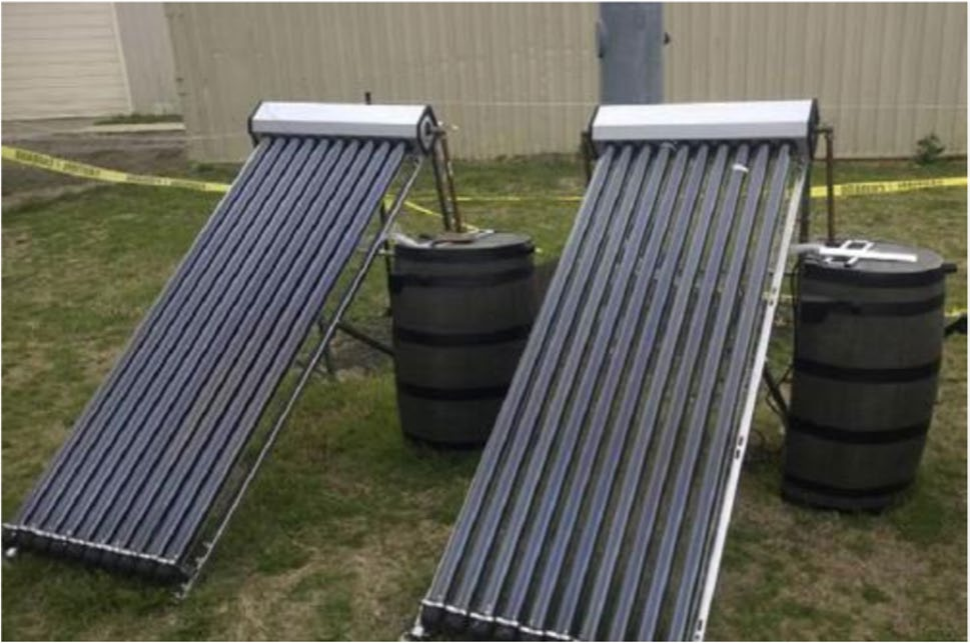
A view of the evacuated tube solar collectors (Papadimitratos et al. 2016)

The supply of these collectors started in the late 1970s, about 70 years after the first use of flat plate collectors. There are various kinds of vacuum tube collectors, in which the absorbent surface is usually surrounded by a double-walled glass tube with a vacuum between the walls. The most significant properties of vacuum tube collectors are the low influence of the sun’s motion during 24 h on the heat flux received by the absorber and the fact that the working fluid inside the collector does not freeze due to cold. Evacuated tube solar collectors are the most appropriate technology solar for generating beneficial heat in both low and medium temperature levels (Kumar et al. 2021a). It can be noted that the installed area of this kind of collector reaches 91,000 m^2^ (Weiss and Mauthner 2010). For instance, a picture of the evacuated tube solar collectors is displayed in Fig. 4.

### Parabolic dish collector (PDC)

**Fig. 5:**
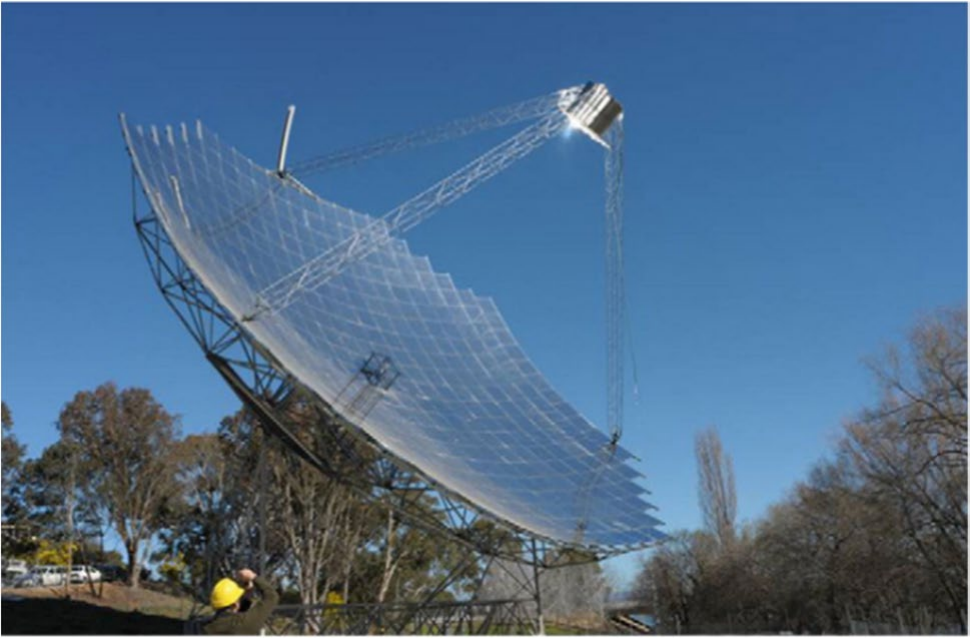
A view of the parabolic dish collectors (Lovegrove et al. 2011)

**Fig. 6:**
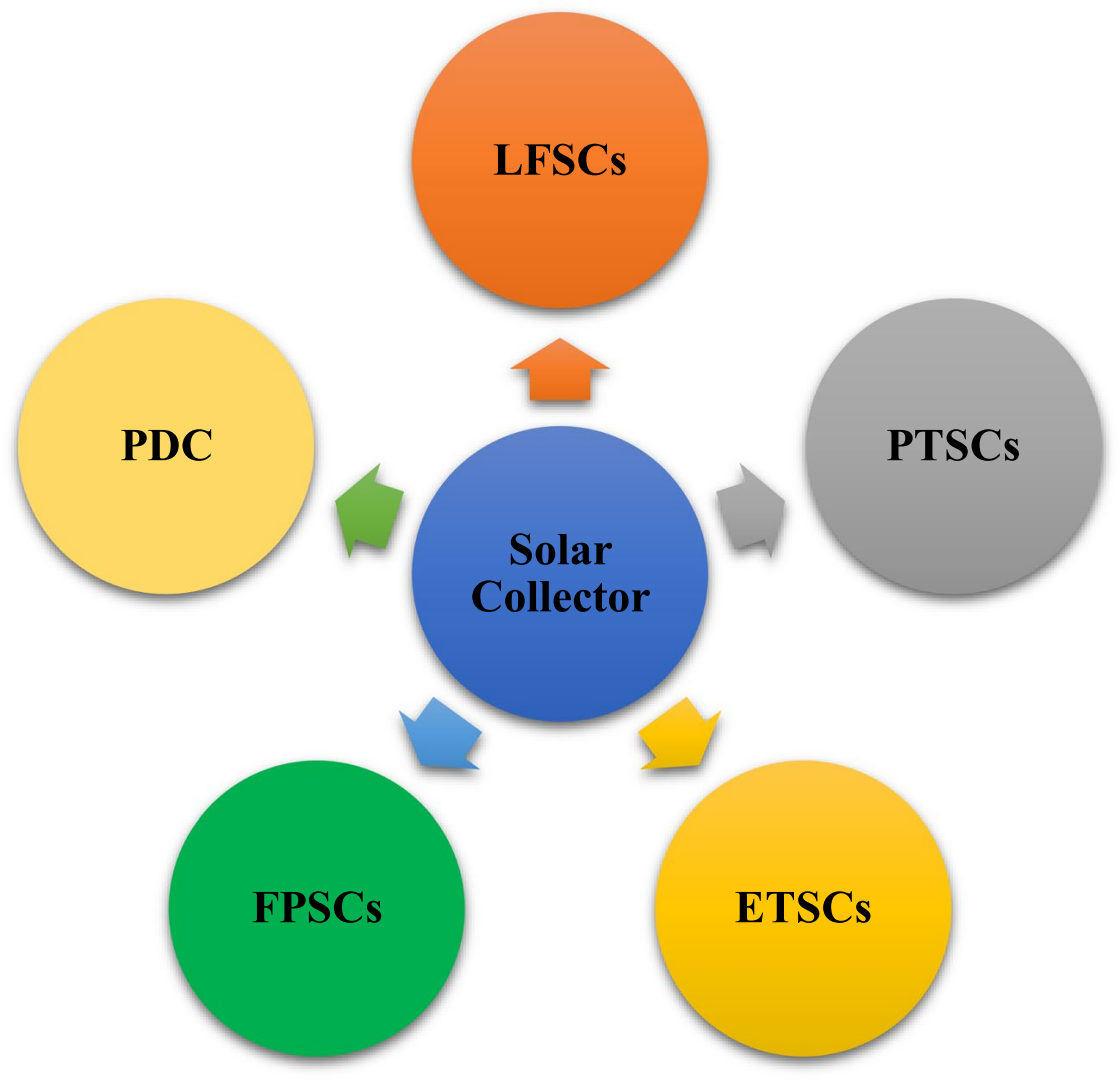
Types of collectors examined in the study

The parabolic dish collector is a type of solar energy system that uses a parabolic-shaped dish to concentrate sunlight onto a receiver located at the focal point of the dish. The collector consists of a large parabolic dish made of reflective material, such as mirrors or shiny metal surfaces. The parabolic shape of the dish allows it to focus incoming sunlight onto a small area at the focal point. Parabolic dish collectors are known for their high concentration ratio, which means they can achieve extremely high temperatures and generate significant power output in a small area. They are particularly suitable for applications requiring high-temperature heat (e.g., solar hydrogen production) or when a concentrated beam of light is needed. (Cherif et al. 2019). For instance, a picture of the parabolic dish collectors is presented in Fig. 5. Also, Fig. 6 is given for easy access to the types of collectors examined in the study.

## Methods of improving heat transfer in FPSCs

Nowadays, new ways are carried out for better heat exchange in variant thermal systems. In this regard, multiple approaches are implemented to increase heat exchange. Based on the literature (Bergles et al. 1983, Bergles et al. 1991), the ways of increasing heat transfer can be divided into three gangs: active, passive, and combined techniques.

### Active methods

The existence of at least one external energy source is the difference between this method and the passive method. This can include surface vibration (Zhou et al. 2022), magnetic or electric field (Giwa et al. 2021, Hamida and Hatami 2021, Izadi et al. 2023a), jet impact (Baghel et al. 2021), suction (Mamori et al. 2021), injection (Jalali et al. 2019), and mechanical aids (Léal et al. 2013).

### Passive methods

Passive techniques deal with changes created in the thermal systems to enhance the thermal efficiency of the systems while no longer requiring external energy sources (Rashidi et al. 2019, Alshuraiaan et al. 2023). Various techniques have been used, containing the utilization of porous materials (Izadi et al. 2019, Peng et al. 2021), microchannel heat sinks (Izadi et al. 2013a, Mehryan et al. 2020b, Lanjwani et al. 2021), inserts (such as twisted strips, coils, swirling flow devices, and turbulators) (Zaboli et al. 2019, Shehzad et al. 2021a, Ajarostaghi et al. 2022, Noorbakhsh et al. 2022, Izadi et al. 2023b), and rough surfaces. For example, wavy surfaces (He et al. 2022), elongated surfaces (such as fins) (Goel and Singh 2021, Shehzad et al. 2021b), depressions and ridges (Cao et al. 2021a), change material (Shehzad et al. 2021a, Izadi et al. 2022a, Xiong et al. 2022), nanofluids (Izadi et al. 2013b, Valipour et al. 2017, Mohammadpour et al. 2022), spiral tubes (Xu et al. 2022), and helical tubes (Rashidi et al. 2021). Some passive methods focused on improving the rate of heat exchange are further discussed in the following.

#### Twisted tapes

Twisted tapes are a type of heat transfer enhancement device used in various industrial applications, particularly in heat exchangers. These kinds of inserts are commonly metalliferous strips that are twisted in some specific shape to form an orderly pattern. The twisted tape, such as a tube or pipe, is typically inserted into the flow passage to enhance heat transfer between the fluid flowing inside the passage and the surrounding walls (Zheng et al. 2017, Gnanavel et al. 2020). Enhanced heat transfer, compact design, energy savings, and versatility can be mentioned among the advantages of twisted tapes. Conical tapes are an example of this method of heat transfer enhancement (Liu et al. 2018; Bahiraei and Gharagozloo 2020).

#### Baffles

Baffles are used as flow-directing panels for liquid or gas flow. By using the baffle, the dead areas are eliminated, and better mixing of the flow in the system is done, and as a result, the heat transfer is improved (Bahiraei et al. 2021, El-Said et al. 2021, Uosofvand and Abbasian Arani 2021). Enhanced heat transfer, flow control, residence time control, and vibration reduction can be mentioned among the advantages of twisted tapes.

#### Winglets and vortex generator

The “wing” portrays the situation when the wing’s dorsal edge is connected to the plate. If the wing’s arch is connected to the end, its name is “winglet.” A vortex generator (VG) is an aerodynamic machine, including a small vane generally connected to a lifting plate. In the attendance of winglets and vortex generators, the resulting rotational flow leads to the appropriate dispensation of temperature in both the longitudinal and radial directions (Zhai et al. 2019, Modi et al. 2020).

#### Wire coil

This sector focuses on using helical or spiral coil tubes to improve thermal efficiency in thermal systems. The use of spiral tubes increases the heat exchange area, resulting in better heat exchange (Alimoradi et al. 2017, Zheng et al. 2018, Saydam et al. 2019, Fadaei et al. 2023).

#### Extended surface (Fin)

A fin is a thin component or appendage attached to a larger body or structure plane that continued from an object to go up the heat exchange rate. A pin fin, a ring fin, and a straight fin with constant and variable areas can be mentioned as types of fins. (Borhani et al. 2019, Gong et al. 2021, Izadi et al. 2023c, Saedodin et al. 2023).

#### Nanofluids

**Fig. 7:**
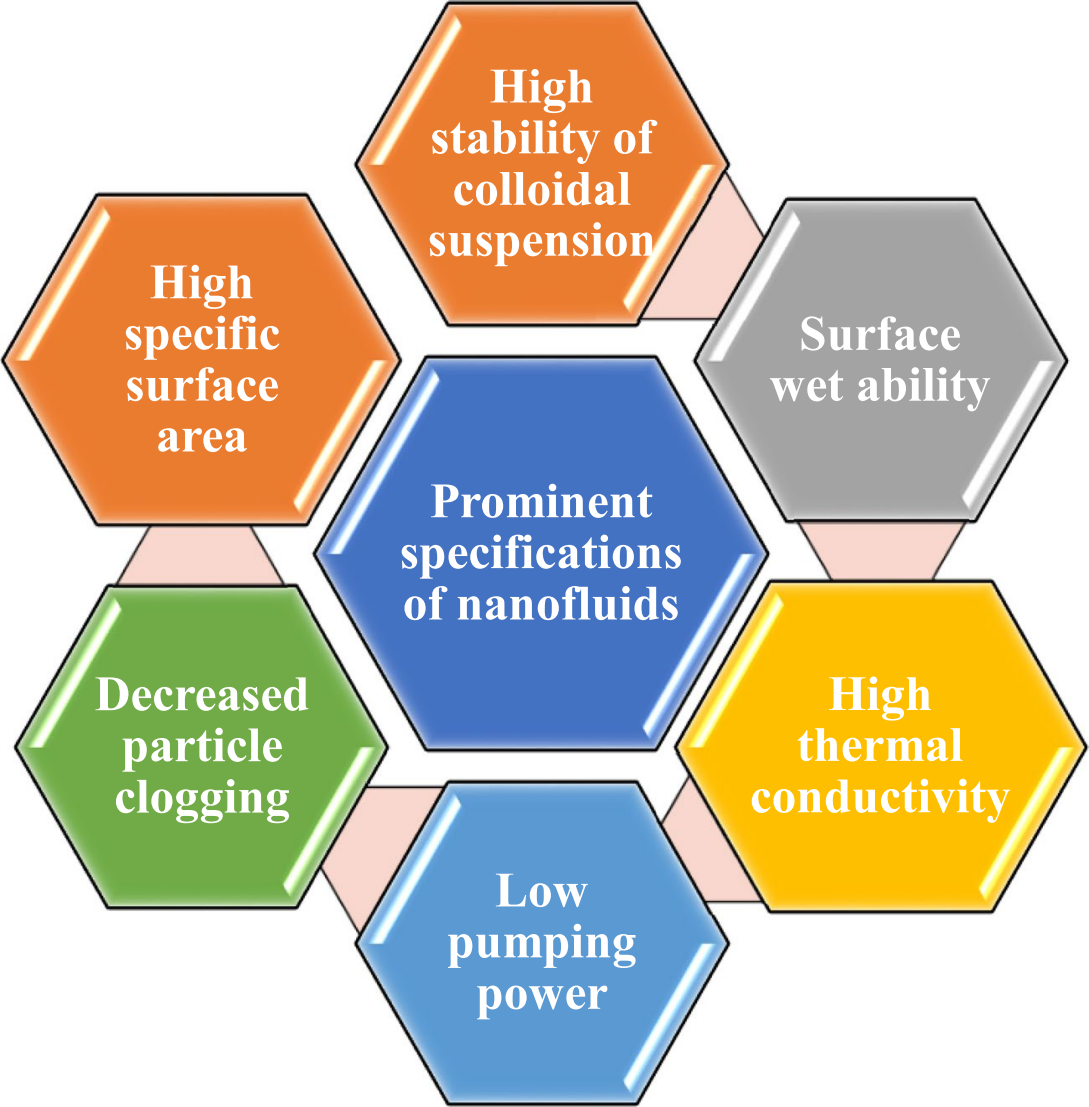
Eminent characteristics of nanofluids

**Fig. 8:**
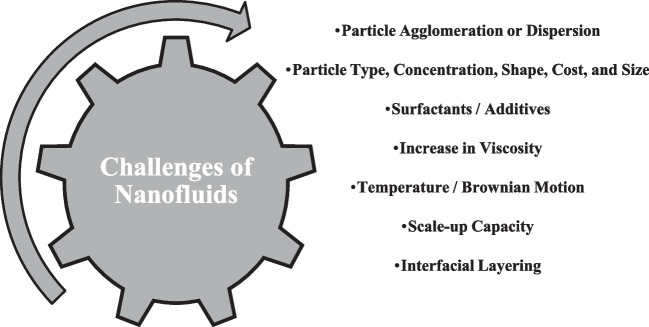
Problems of using nanofluids

With worldwide competition in the field of different industries and the importance of energy in the cost of production, these industries are intensely moving towards developing new and advanced fluids with high thermal indices. Nanotechnology is one of the factors of progress in various industries. Nanotechnology involves a series of activities at the nanometer scale. One of the fields of action of this new technology is the production of particles with nanometer dimensions (nanoparticles). Among the applications of nanoparticles, we can mention the increase in thermal and chemical resistance and improvement in the strength of the produced materials. The nanoparticles’ high surface-to-volume ratio is another one of the properties of this material (Izadi 2020). Conforming to this feature, strong catalysts can be made on the nanoscale. Nanofluids are manufactured of stable carbon suspensions with high thermal conductivity, based on metal, and non-metal, which are suspended in fluids called base fluids such as glycol, oil, acetone, water, and ethylene (Buongiorno 2006, Taylor et al. 2013, Izadi et al. 2018). Nanofluids, a cutting-edge category of fluids, have garnered significant attention in research circles. Increasing evidence suggests that nanofluids outperform traditional fluids in diverse heat transfer applications (Mehryan et al. 2020a). In 1995, Choi from the Energy Technology Department of the Argonne National Laboratory of the United States first proposed the issue of nanofluid as a new environment for heat exchange. Recently, many researchers checked the influence of nanofluid applications and the alteration of the thermophysical properties of these fluids on different devices. These properties consist of specific heat capacity, density, adhesion force, thermal conductivity coefficient, viscosity, etc. (Choi and Eastman 1995). Eminent characteristics and some problems with using nanofluids of nanofluids are shown in Figs. 7 and 8.

#### Models of thermo-physical properties for mono nanofluids and hybrid nanofluids

There are generally two single-phase and two-phase models in the modeling process of nanofluid flow in FPSCs. In the first one, the base fluid and nanoparticle are formed into a single-phase fluid, and thermos-physical properties varying with temperature have been presented according to the experimental outcomes of various research (Izadi and Assad 2021, Xiong et al. 2021a, Xiong et al. 2021b). The interaction between the two steps is considered in the two-phase model, where the nanoparticle is in the solid phase, and the base fluid is in the liquid phase. Among the two considered standards, the most common model for nanofluid flow modeling is the single-phase model, whose advantages are the simplicity of the equations and, consequently, the decline in cost and simulation time (Izadi et al. 2015a). Furthermore, the results presented in related works have shown that the single-phase model has acceptable precision. The main challenge in using single-phase models for nanofluid modeling is the use of appropriate temperature-dependent experimental correlations for different thermos-physical properties of nanofluids.

Assuming nanofluids to homogenized (single phase) mixtures, the following equations are often used to approximate the thermophysical properties (Izadi et al. 2015b, Hu et al. 2021, Sajjadi et al. 2021, Yang et al. 2021, Kazaz et al. 2022):

1 $${\rho }_{nf}=\phi {\rho }_{np}+(1-\phi ){\rho }_{bf}$$

2 $${k}_{nf}={k}_{bf}\left[\frac{{k}_{bf}+{k}_{np}+n{k}_{bf}+\phi ({k}_{np}-{k}_{bf})-n\phi ({k}_{bf}-{k}_{np})}{{k}_{bf}+{k}_{np}+n{k}_{bf}+\phi ({k}_{bf}-{k}_{np})}\right]$$

3 $$({C}_{p}{)}_{nf}=\frac{\phi (\rho {C}_{p}{)}_{np}+(1-\phi )(\rho {C}_{p}{)}_{bf}}{{\rho }_{nf}}$$

4 $${\mu }_{nf}=\frac{{\mu }_{bf}}{(1-\phi {)}^{2.5}}$$

If two or more nanoparticles are used in the nanofluid instead of one type of nanoparticle, the obtained nanofluid is a hybrid type. These models for hybrid nanoparticles take the following form (Li et al. 2020, Javadi et al. 2021, Asadi et al. 2022, Mousavi Ajarostaghi et al. 2022).

5 $${\rho }_{hnf}={\phi }_{np1}{\rho }_{np1}+{\phi }_{np2}{\rho }_{np2}+(1-{\phi }_{np1}-{\phi }_{np2}){\rho }_{bf}$$

6 $$({C}_{p}{)}_{hnf}=\frac{{\phi }_{np1}(\rho {C}_{p}{)}_{np1}+{\phi }_{np2}(\rho {C}_{p}{)}_{np2}+(1-{\phi }_{np1}-{\phi }_{np2})(\rho {C}_{p}{)}_{bf}}{{\rho }_{hnf}}$$

7 $${\mu }_{hnf}=\frac{{\mu }_{bf}}{(1-{\phi }_{np1}-{\phi }_{np2}{)}^{2.5}}$$

8 $${k}_{hnf}=\frac{2\left({\phi }_{np1}{k}_{np1}+{\phi }_{np2}{k}_{np2}\right)-2{k}_{bf}\left({\phi }_{np1}+{\phi }_{np2}\right)+2{k}_{bf}+\left[\frac{{\phi }_{np1}{k}_{np1}+{\phi }_{np2}{k}_{np2}}{{\phi }_{np1}+{\phi }_{np2}}\right]}{-\left({\phi }_{np1}{k}_{np1}+{\phi }_{np2}{k}_{np2}\right)-{k}_{BF}\left({\phi }_{np1}+{\phi }_{np2}\right)+2{k}_{bf}+\left[\frac{{\phi }_{np1}{k}_{np1}+{\phi }_{np2}{k}_{np2}}{{\phi }_{np1}+{\phi }_{np2}}\right]}$$

### Combined methods

In a combined method, two or more active and passive methods are used together to improve the system’s thermal efficiency, producing a higher heat exchange rate than individually provided by either technique. By combining passive and active methods, it is possible to achieve synergistic effects and maximize the overall heat transfer performance. For example, passive methods can create an optimized heat transfer environment. In contrast, active methods can provide additional control and adjustability to match specific heat transfer requirements or accommodate varying operating conditions. Factors such as cost-effectiveness, system complexity, energy consumption, and available resources should be considered when selecting and integrating passive and active methods for increasing heat transfer efficiency. (Valipour et al. 2018, Izadi et al. 2020, Saedodin et al. 2020, Saedodin et al. 2021, Alshuraiaan et al. 2022, Izadi et al. 2022b, Mashayekhi et al. 2022, Shang et al. 2022).

## Flat plate solar collectors with nanofluids

Some recent research focusing on ways to better the rate of heat transfer with nanofluid in flat plate collectors is mentioned below. In 2012, Yousefi et al. (Yousefi et al. 2012) experimentally checked the impact of aluminum nanofluid on the performance of an FPSC. In this study, the ASHRAE standard was used to compute efficiency. The outcomes displayed that compared to water as the absorption medium, the usage of nanofluid as the base fluid increases the efficiency. For example, in 0.2% by weight, the efficiency increase was 28.3%. Pressure drop and heat transfer of an absorbent medium with suspended nanoparticles (aluminum oxide, copper oxide, titanium dioxide, and silicon dioxide dispersed in water) inside a flat plate solar collector were reviewed by Alim et al. (Alim et al. 2013). Based on the analytic outcomes, the copper oxide nanofluid average Nusselt number increased by 22.15% compared to base fluid as an absorbent fluid and reduced the entropy production by 4.34%. In 2014, Said et al. (Said et al. 2014) investigated entropy generation, heat transfer improvement, and pressure drop capabilities for a flat plate solar collector with nanofluid-based single-walled carbon nanotubes (SWCNTs). According to the report, the single-walled carbon nanotubes nanofluid decreased the entropy production by 4.34%, and the pumping power of the nanofluid solar collector was 1.20% higher than the base fluid. Safarian et al. (Saffarian et al. 2020) assessed the increase of heat transfer in a flat plate collector using nanofluids in different concentrations. For investigating the changes in the average Nusselt number in the pipes, numerical simulations were performed at speeds of 0.5, 1, 2, and 4 m/s. The results showed that adding nanoparticles caused an increment in the heat transfer coefficient. Gupta et al. (Gupta et al. 2021) checked the performance of flat plate solar collectors with and without nanofluid containing aluminum oxide nanoparticles. The water temperature at the flat plate solar collector outlet without nanofluid was 5–10 °C lower than when the nanofluid was used. Sundar et al. (Sundar et al. 2020a) experimentally checked the energy performance, economic influence, and heat exchange aspects of solar flat plate collectors using aluminum oxide nanoparticles. Based on the reported outcomes, using nanofluid increased the collector’s efficiency by 20%. Some recent research focusing on ways to ameliorate the heat transfer rates with nanofluid in flat plate collectors are listed in Table 1.

**Table 1:** Overview on ways to ameliorate the heat transfer rates with nanofluid in flat plate collectors

Author	Type of study	Base fluid	Nanoparticle	Concentration	Remarks
He et al. (He et al. 2015)	Experimental	Water	Cu	0.1 0.2	Also, the highest temperature and the highest go up in base fluid temperature in the nanofluid tank compared to the water tank went up to 12.24% and 24.52%, respectively
Meibodi et al. (Choudhary et al. 2021)	Experimental	Ethylene glycol (EG)–water	SiO_2_	0.5 0.75 1	The outcomes illustrated that the nanofluid led to an increase in efficiency of approximately 4 to 8%
Shojaeizadeh et al. (Shojaeizadeh et al. 2015)	Experimental	Water	Al_2_O_3_	0.0906_ 0.1423	By suspending the nanoparticles in water, the respective optimal collector inlet fluid temperature and fluid mass flow values decreased by about 2% and 68%, respectively
Colangelo et al. (Colangelo et al. 2015)	Experimental	Water	Al_2_O_3_	3%	Experimental outcomes displayed that the thermal efficiency increased to 11.8% compared to the base fluid measured using nanofluids
Said et al. (Said et al. 2015b)	Numerical	Water	SWCNTs	0.1 0.3	By improving the thermophysical properties of the nanofluid, the maximum energy efficiency and exergy of the flat plate collector were obtained up to 95.12% and 26.25%
Michael and Iniyan (Michael and Iniyan 2015)	Experimental	Water	CuO	0.05	According to the reported results, the thermal performance of the solar water heater improved by up to 6.3%
Sabiha et al. (Sabiha et al. 2015)	Experimental	Water	SWCNTs	0.05 0.1 0.2	Collector efficiency with nanofluid was reported to be 71.84% higher than pure water
Said et al. (Said et al. 2015a)	Experimental	Water	TiO_2_	0.1 0.3	Using nanofluid, the thermal conductivity increased up to 6%. Also, exergy efficiency and energy efficiency improved by 16.9% and 76.6%, respectively
Verma et al. (Verma et al. 2016)	Experimental	Water	MgO	0.25 0.5 0.75 1 1.25 1.5	Experimental observations showed an increase in thermal efficiency and exergetic efficiency of 9.34% and 32.23%, respectively
Said et al. (Said et al. 2016a)	Experimental	Water	Al_2_O_3_	0.1	According to the results obtained in the nanoparticle size study, the highest energy and exergy efficiency were 73.7% and 20.3%, respectively
Vincely and Natarajan (Vincely and Natarajan 2016)	Experimental	Water	Graphene oxide	0.02	The maximum efficiency of solar collectors went up by 11.5%
Salavati et al. (Meibodi et al. 2016)	Experimental	Ethylene glycol–water	SiO_2_	0.5 0.75 1	Exergy efficiency at the highest tested concentration had its highest value this increase reached 62.7%
Said et al. (Said et al. 2016b)	Experimental	Water	Al_2_O_3_	0.1 0.3	The outcomes indicated that energy efficiency improved by 83.5%
Ahmadi et al. (Ahmadi et al. 2016)	Experimental	Water	Graphene nanoplatelets	0.01 0.02	Conforming to the results, the dispersion of graphene in the water increased the thermal efficiency by 18.87%
Verma et al. (Verma et al. 2017)	Experimental	Water	Al_2_O_3_, SiO_2_, TiO_2_, CuO, graphene	0.75	The results show that the highest exergy and energy efficiencies recorded for carbon nanotubes were reported by 29.32% and 23.47%, respectively
Sharafeldin et al. (Sharafeldin et al. 2017)	Experimental	Water	WO_3_	0.016 0.0333 0.0666	The highest efficiency of solar collectors went up by 13.48%
Edalatpour and Solano (Edalatpour and Solano 2017)	Numerical	Water	Al_2_O_3_	1 2 3 4 5	The heat transfer coefficient raised from 10 to 65% despite the nanofluid
Sint et al. (Sint et al. 2017)	Experimental	Water	CuO	0.1 0.5 1 2	The nanofluid usage as a general fluid improved the collector efficiency by up to 5% in environmental conditions
Kang et al. (Kang et al. 2017)	Experimental	Water	Al_2_O_3_	0.5 1 1.5	Heat efficiency was reported to be 74.9%, which was 14.8% higher than base fluid
Jouybari et al. (Jouybari et al. 2017)	Experimental	Distilled water	SiO_2_	0.2 0.4 0.6	Based on the experimental data, the thermal efficiency was enhanced up to 8.1% in the nanofluid stream
Kim et al. (Kim et al. 2017)	Experimental	Water	Al_2_O_3_	1	The results depicted that the maximum efficiency was 24.1% higher than base fluid use, and the highest efficiency was 72.4%
Tahat et al. (Tahat and Benim 2017)	Experimental	Ethylene glycol and water	Al_2_O_3_/CuO	0.5 1 1.5 2	The results showed that thermal conductivity, viscosity, and density went up with nanoparticle concentration. Also, Collector efficiency improved by up to 45% in the presence of composite nanoparticles
Bianco et al. (Bianco et al. 2018)	Numerical	Water	Al_2_O_3_	2 4 6	The average Nusselt number and the average heat transfer coefficient for nanofluids increased in the range of 2 to 15%
Sharafeldin et al. (Sharafeldin and Gróf 2018)	Experimental	Water	CeO_2_	0.0167 0.0333 0.0666	According to the results, the efficiency of solar collectors improved by 10.74%
Farajzadeh et al. (Farajzadeh et al. 2018)	Experimental numerical and	Water	Al_2_O_3_/TiO_2_	0.1	Experimental results showed that the thermal efficiency increased by 26% using a mixture of two nanofluids
Genc et al. (Genc et al. 2018)	Numerical	Water	Al_2_O_3_	1 2 3	According to the results, the nanofluid increased the output temperature by 7.20% compared to water
Kiliç et al. (Kiliç et al. 2018)	Experimental	Water	TiO_2_	2	The highest efficiency for nanofluids was 48.67%, while the highest efficiency for pure water was 36.20%
Mirzaei et al. (Mirzaei et al. 2018)	Experimental	Water	Al_2_O_3_	0.1	The average efficiency was elevated by 23.5% compared to pure water as a working fluid
Kashyap et al. (Kashyap et al. 2018)	Numerical	Ethylene glycol and water	ZnO	0.02	The outcomes indicated a 39.17% increase in exergy efficiency when using nanofluids
Hawwash et al. (Hawwash et al. 2018)	Experimental Numerical	Water	Al_2_O_3_	0.1 0.5 1 2 3	The use of nanofluids improved the efficiency by about 18.3%
shamshirgaran et al. (Shamshirgaran et al. 2018)	Numerical	Water	Cu	1 2 3 4	The results showed that exergy efficiency and energy efficiency increased by 10.5% and 8%, respectively
Ameri et al. (Ameri and Eshaghi 2018)	Experimental	Water	Fe_3_O_4_	1 2	The outcomes displayed that the system’s thermal efficiency went up to 83.97%
Purohit et al. (Purohit et al. 2018)	Numerical	Water	Al_2_O_3_	1 4 6	The simulation results showed a 25.2% betterment in the heat transfer coefficient by the nanofluid
Bazdidi-Tehrani et al. (Bazdidi-Tehrani et al. 2018)	Numerical	Water	CuO and TiO_2_	0.99 2.04	Using nanofluids, the efficiency of the solar collector increased by approximately 10%
Ziyadanogullari et al. (Ziyadanogullari et al. 2018)	Experimental	Water	Al_2_O_3_, CuO, and TiO_2_	0.2 0.4 0.8	The maximum improvement in solar collector performance obtained 63.71%
Bellos and Tzivanidis (Bellos and Tzivanidis 2018)	Experimental	Water	Cu	2	The final optimal system with nanofluid provided a 3.99% increase in exergy
Sundar et al. (Sundar et al. 2018b)	Experimental	Water	Al_2_O_3_	0.1 0.3	Simple collector efficiency for nanofluid increased from 0.3% to 58%
Verma et al. (Verma et al. 2018)	Numerical	Water	Hybrid CuO and MgO with MWCNTs	0.25 0.5 0.75 1 1.25 1.5 1.75 2	Percentages of increase in energy efficiency and collector energy for hybrid nanofluids were 25.1% and 16.28%
Sundar et al. (Sundar et al. 2018a)	Experimental	Water	Al_2_O_3_	0.5 1 1.5	The maximum improvement in solar collector performance was 24.1%. In addition, the thermal efficiency went up by 18%
Tang et al. (Tong et al. 2010, 2019)	Experimental	Water	Al_2_O_3_/CuO	0.5 1	The solar collector’s thermal efficiency using nanofluid has been increased by 16%
Eltaweel et al. (Eltaweel and Abdel-Rehim 2019)	Experimental	Distilled Water	MWCNTs	0.01 0.05 0.1	Experimental outcomes indicated that the maximum exergy efficiency for a flat panel solar collector using carbon nanotubes was approximately 23.35%, which increased by approximately 9%
Akram et al. (Akram et al. 2019)	Experimental	Water	Eco-friendly treated graphene nanoplatelets	0.025 0.075 0.1	The highest thermal efficiency with the use of additives reached 78%
Michael et al. (Stalin et al. 2019)	Experimental	Water	CeO_2_	0.01	The outcomes indicated that the maximum efficiency with nanofluid is 78.2%, which is 21.5% higher than water as a base fluid
Asker and Gadanya (Asker and Gadanya 2019)	Numerical	Water	Al_2_O_3_, CeO_2_, Cu, SiO_2_, and TiO_2_	2	According to the results, the maximum increase in efficiency in SiO_2_ nanofluid is reported to be 10%
Gangadevi and Vinayagam (Gangadevi and Vinayagam 2019)	Experimental	Water	Hybrid nano (Al_2_O_3_/CuO)	0.05 0.1 0.2	According to the results, the maximum increase in efficiency in SiO_2_ nanofluid was reported by 10%. Also, the maximum electrical and thermal efficiencies of the solar collector were 15% and 82% of the hybrid nanofluids at the peak of solar radiation, respectively
Alawi et al. (Alawi et al. 2019)	Experimental	Pentaethylene glycol	Treated graphene nanoplatelets	0.025 0.05 0.075 0.1	According to the outcomes, the collector efficiency used with the nanofluid went up by a maximum of 13.3% compared to water
Rajput et al. (Rajput et al. 2019)	Experimental	Water	Al_2_O_3_	0.1 0.2 0.3	The outcomes displayed that with increasing the volume fraction, a 21.32% go up in collector efficiency was observed
Hussein et al. (Hussein et al. 2020)	Experimental	Distilled water	Hexagonal boron nitride (h-BN)-graphene nanoplatelets (CF-GNPs) with covalent functionalized-multi wall carbon nanotubes (CF-MWCNTs)	0.05 0.08 0.1	Solar collector efficiency has been got up by up to 85% with hybrid nanofluids. In addition, raising the concentration of nanoparticles increases the thermal energy and temperature of the fluid outlet
Moravej et al. (Moravej et al. 2020)	Experimental	Water	TiO_2_	1 3 5	According to the results shown, with increasing solar radiation, the impact of adding nanoparticles increased so that the maximum collector efficiency when using nanofluid was reported to be approximately 78%
Aghili and Kasaeian (Aghili Yegane and Kasaeian 2020)	Numerical	Water	Hybrid nano (Al_2_O_3_/CuO)	0.05 0.1	The thermal efficiency went up using a combined nanofluid
Lee et al. (Lee et al. 2020)	Experimental	Water	MWCNT/Fe_3_O_4_	0.003 0.005 0.01 0.015	The maximum efficiency was 80.3% for hybrid nanofluids, which was 17.6% higher than the base fluid
Munuswamy et al. (Munuswamy et al. 2020)	Experimental and numerical	Water	Al_2_O_3_/CuO	0.2 0.4	The results depicted that the use of nanofluid went up the efficiency in the collector by 2.16%
Choudhary et al. (Choudhary et al. 2020a)	Experimental	Water	ZnO	1%	According to the given outcomes, the maximum thermal efficiency is reported to be 69.24%
Meibodi et al. (Meibodi et al. 2015)	Numerical	Water	Al_2_O_3_, TiO_2_ SiO_2_, polystyrene, GNP, SWCNT	0.04 0.05 0.06 0.07 0.07 0.1 0.2 0.3 0.4 1 2 3 4	The results show that the collector efficiency increased with increasing nanofluid concentration
Sundar et al. (Sundar et al. 2020b)	Experimental	Water	Based nanodiamond	0.2 0.4 0.6 0.8 1	According to the outcomes, the heat transfer and average Nusselt number heat transfer increased by 52.33% and 32.31%, respectively
Sharma et al. (Sharma et al. 2020)	Experimental	Water	CeO_2_	0.25 0.5 0.75 1 1.25 1.5 1.75 2	The maximum collector efficiency increased to 57.1%
Choudhary et al. (Choudhary et al. 2020b)	Experimental	Ethylene glycol-distilled water	MgO	0.08 0.14 0.2 0.4	Collector thermal efficiency increased by 16.36% with nanofluid compared to distilled water and ethylene glycol. Also, the absorbed energy parameter is improved by 16.74% compared to the ethylene glycol-distilled water
Choudhary et al. (Sarsam et al. 2020)	Experimental	Triethanolamine	Graphene nanoplatelets	0.025 0.05 0.075 0.1	Observations indicated that by using nanofluid in the collector, the efficiency went up to 10.53%
Sundar et al. (Sundar et al. 2020b)	Experimental	Water	Cu	0.1 0.3	The efficiency with base fluid is 52% and for nanofluids, it increased to 58%
Okonkwo et al. (Okonkwo et al. 2020)	Experimental	Water	Hybrid nanofluids, Al_2_O_3_, Fe_3_O_4_	0.05 0.1 0.2	The results illustrate that the use of nanofluid increased the heat in the collector by 2.16%
Michael et al. (Stalin et al. 2020)	Experimental	Water	CeO_2_	0.01 0.05 0.1	According to the results, the efficiency of the solar collector when using nanofluids is 28.07% higher than water
Alzahrani et al. (Alzahrani et al. 2021)	Experimental	Water	Hybrid nanofluid (MgO, CuO, MWCNTs	0.01 0.02 0.003	Observations showed that nanoparticle volume fraction boosts solar radiation's absorption and transmission efficiency
Farhana et al. (Farhana et al. 2021)	Experimental	Water	Al_2_O_3_, crystal nano-cellulose (CNC)	0.1 0.3 0.5	Aluminum oxide and crystal nano-cellulose increased the collector performance by 2.48% and 8.46%, respectively
Darbari and Rashidi (Darbari and Rashidi 2021)	Numerical	Water	Cu, CuO	0.01 0.02 0.03 0.04 0.05	Adding copper and copper oxide nanoparticles with a volume fraction of 5% to the base fluid increased the solar collector energy by 6% and 3%, respectively
Xiong et al. (Xiong et al. 2021a)	Numerical	Water	Hybrid nanofluid Ag-Al_2_O_3_	0.1	The use of hybrid nanofluids increased the collector performance
Allouhi and Amine (Allouhi and Amine 2021)	Numerical	Water	CuO, Al_2_O_3_ and TiO_2_	1 2 3	Maximum energy efficiency and exergy improvements were reported for CuO-based nanofluids 2.7 and 11.1, respectively
Akram et al. (Akram et al. 2021)	Experimental	Water	Graphene nanoplatelets, ZnO, SiO_2_	0.025 0.05 0.075 0.1 0.15 0.2	The outcomes displayed that the highest growth in thermal conductivity of nanofluids increased to 25.68%
Alklaibi et al. (Alklaibi et al. 2021)	Experimental	Distilled water	Nanodiamond	0.2 0.4 0.6 0.8 1	Experimental outcomes displayed that the highest collector thermal efficiency was 69.85%, 12.7% higher than pure water
Gad et al. (Gad et al. 2021)	Experimental	Water	Al_2_O_3_ and TiO_2_	2	The maximum increase for aluminum and titanium oxide was about 22% and 30%, respectively, compared to the water
Bezaatpour and Rostamzadeh (Bezaatpour and Rostamzadeh 2021a, 2021b)	Numerical	Water	Fe_3_O_4_	2	The results demonstrated that the energy and exergetic performance of the collector increased by 5.83% and 3.21%, respectively
Mustafa et al. (Mustafa et al. 2021)	Numerical	Water	CuO	0.1	Solar process efficiency improved by 12.8%
Kumar et al. (Kumar et al. 2021b)	Experimental	Water	Graphene	0.025 0.05 0.1	Based on the data, the thermal efficiency increased up to 24%
Eltaweel et al. (Eltaweel et al. 2021)	Experimental	Water	MWCNT	0.005 0.01 0.05	The results showed that the average Nusselt number of solar collectors increased by nearly 48%
Bezaatpour and Rostamzadeh (Bezaatpour and Rostamzadeh 2021a, 2021b)	Numerical	Water	Fe_3_O_4_	0.2	Collector energy efficiency increased from 44.4% to 61.7%. Additives also save 31% on energy waste
Sundar et al. (Sundar et al. 2021)	Experimental	Water	Cu	0.1 0.3	The outcomes illustrated that the increase in nanofluid thermal conductivity compared to water reached 23.56
Khetib et al. (Khetib et al. 2022)	Numerical and experimental	Water	Hybrid nanofluid (DWCNTs-TiO_2_)	1 2 3	The outcomes illustrated that the average Nusselt number increased by 63.46%
Saleh et al. (Saleh et al. 2022)	Experimental	Water	Al_2_O_3_	0.1 0.2 0.3	The outcomes indicated that the collector’s thermal efficiency with water was 53%, while it increased to 65% despite the nanofluid. Also, the average Nusselt number went up to 23.22%
Kumar et al. (Kumar et al. 2022	Experimental	Distilled water	Bio-functionalized graphene	0.025 0.05 0.1	The outcomes illustrated that the maximum increase in thermal efficiency reached 21.48%, and the maximum increase in pressure drop and pumping power was about 0.85% and 0.567%
Mahamude et al. (Mahamude et al. 2022)	Experimental	Water	Hybrid nanofluids (graphene/waste cotton)	0.1 0.3 0.5	The outcomes displayed that the highest efficiency obtained with the combined nanofluids was 16.88% higher than the base state
Ashour et al. (Ashour et al. 2022)	Numerical	Water	ZnO and CuO	0.05 0.1 0.15	The results showed that the best achievement using nanofluids with average efficiency reached 81.64%
Nabi et al. (Nabi et al. 2022)	Numerical	Water	Hybrid nanofluids MWCNT and SWCNT-CuO	1 3 5	The outcomes showed that the average Nusselt number went up by 8.79%
Suthahar et al. (Suthahar et al. 2022)	Numerical	Water	Al_2_O_3_	0.1 0.3 0.5	The average instantaneous thermal efficiency of the collector with nanofluid has reached 84.85%
Stalin et al. (Suthahar et al. 2022)	Numerical	Water	Hybrid nanofluids (Zn-Fe_2_O_4_)	0.02 0.05 0.1 0.2 0.5	The maximum energy efficiency of the collector with a hybrid nanofluid has reached 80.1%

### Type of study using nanofluids in FPSCs

**Fig. 9:**
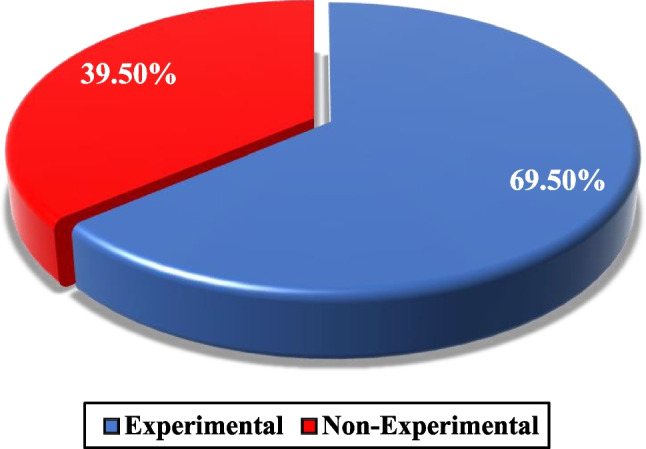
Breakdown of the type of analysis about employing nanofluids in flat plate solar collectors

In recent years, several kinds of research have been conducted on flat plate collectors with different nanofluids. These researches include experimental and non-experimental studies. As shown in Fig. 9, experimental studies comprise the majority of these studies (69.5%). It can also be seen that in the last eight years, non-experimental studies played a more diminutive role in this review, with nearly 40%.

### Type of the base fluid of the employed nanofluids in FPSCs

**Fig. 10:**
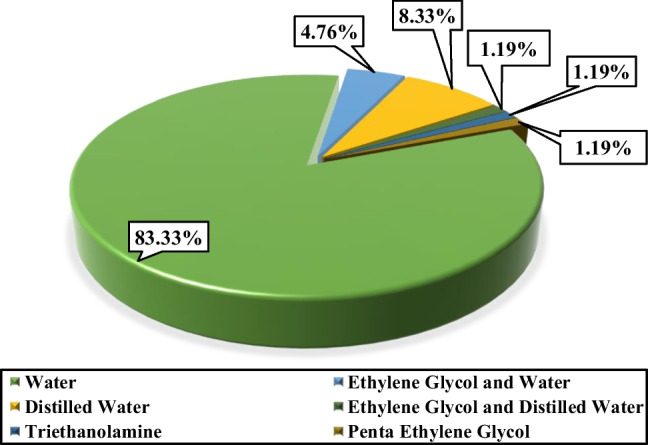
Breakdown of the use of various working fluids in flat plate collector

The working fluid used considerably affects the efficiency of flat plate collectors and other collectors (Xiong et al 2021c), so the different types of working fluids are explained in this section. Since the base fluid plays a considerable role in FPSCs as a heat carrier, paying attention to factors such as avoiding excessive viscosity in the solution, heat capacity, etc., to choose the working fluid is necessary. The results show that storing and recovering more thermal energy by water is possible compared to other base fluids. Figure 10 illustrates the distribution of the usage of several kinds of carrier fluid applied to flat plate collectors. Water is often utilized as the working fluid in the collector (83.33%).

### Type of the nanoparticle of the employed nanofluids in FPSCs

**Fig. 11:**
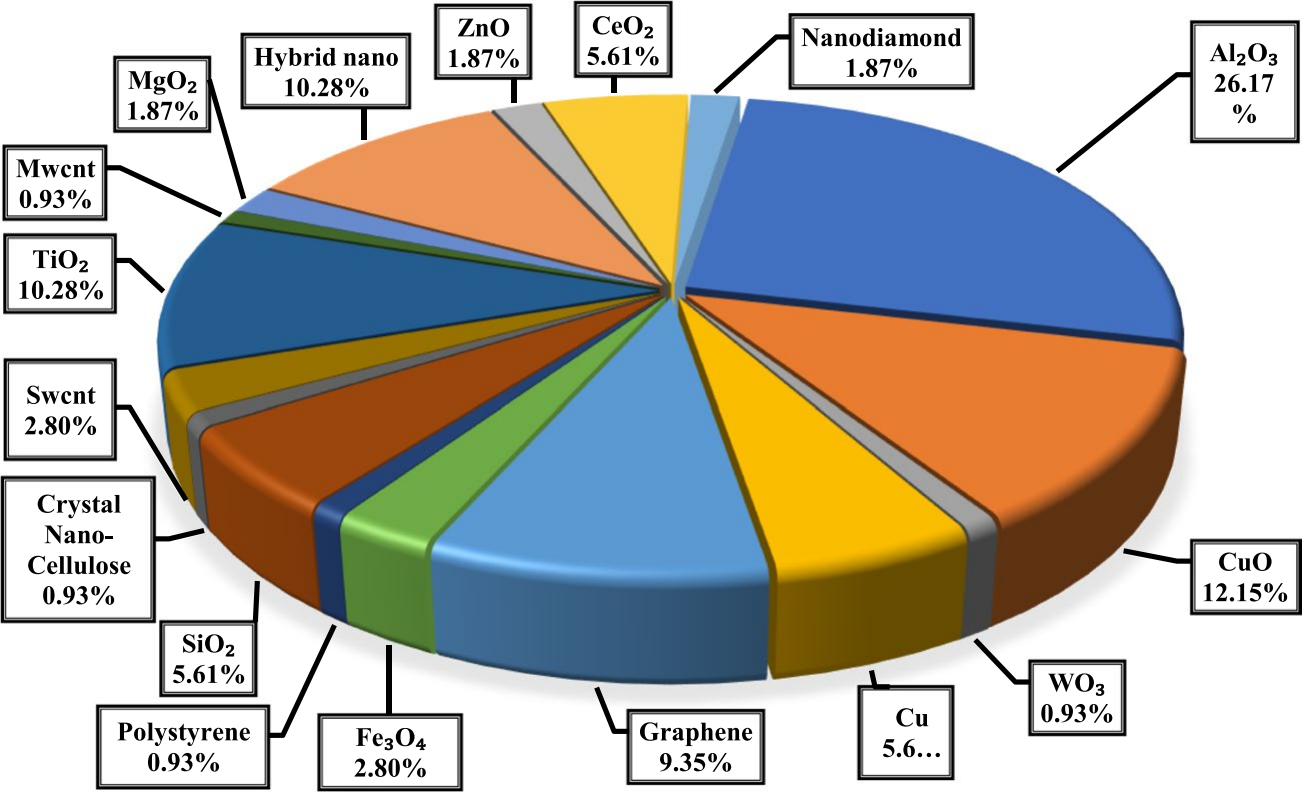
Breakdown of the various nanofluids usage in flat plate collector

Figure 11 shows a usage breakdown of several kinds of nanoparticles applied in the flat plate collectors for 8 years. It is observed that Al_2_O_3_ is often used as a nanoparticle in flat plate collectors (26%), and after that, CuO is in second place (12%). It should also be noted that the number of investigations on using combined nanofluid in FPSCs is relatively high during the last 8 years (about 10%).

### Volume concentration of employed nanofluids in FPSCs

**Fig. 12:**
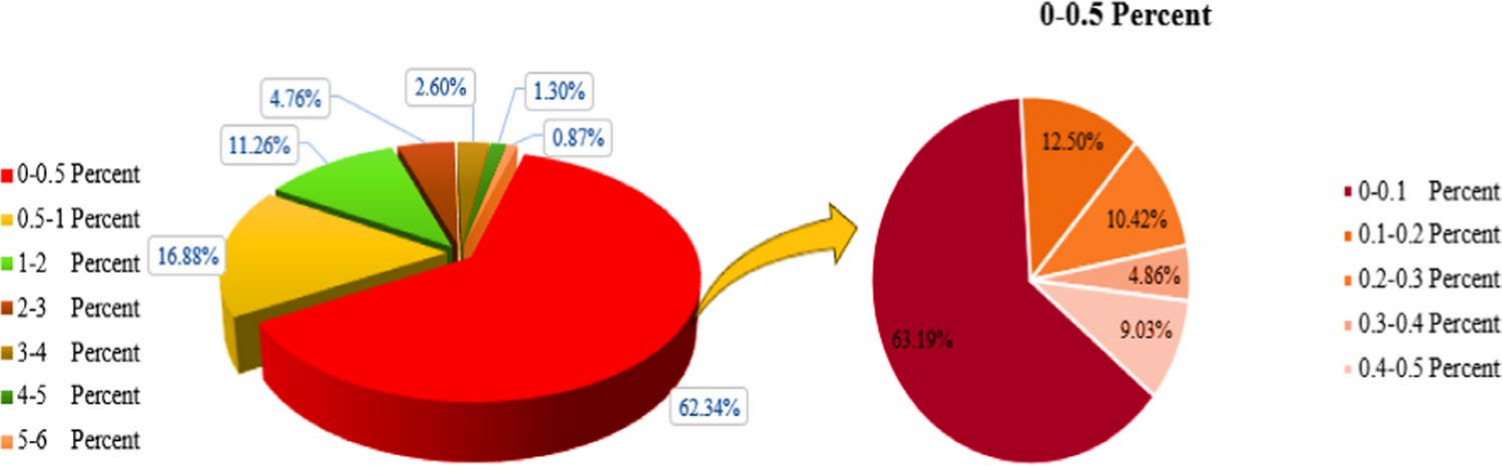
Distribution of the usage of various volume concentrations of nanofluids in FPSCs

Figure 12 illustrates the distribution of the use of various volume concentrations of nanofluids applied to FPSCs. Accordingly, it can be seen that about 62.34% of the works employed nanofluids with the volume concentration in the range of 0–0.5% in which 63.19, 12.5, 10.42, 9.03, and 4.86% of the works belong to the cases with the volume concentration range of 0–0.1%, 0.1–0.2%, 0.2–0.3%, 0.4–0.5%, and 0.3–0.4%, respectively. Furthermore, based on the plotted data in Fig. 12, it can be concluded that about 16.88, 11.26, 4.76, 2.6, 1.3, and 0.87% of the studies evaluated the usage of nanofluids with the volume concentration in the range of 0.5–1, 1–2, 2–3, 3–4, 4–5, and 5–6%, respectively.

### Thermal efficiency of FPSCs utilizing nanofluids

Different assessments have been conducted to facilitate the thermal performance of flat plate solar collectors. This study aims to assess the current two methods, including nanofluids and inserts (enhancement devices), to improve the thermal performance of FPSC.

**Fig. 13:**
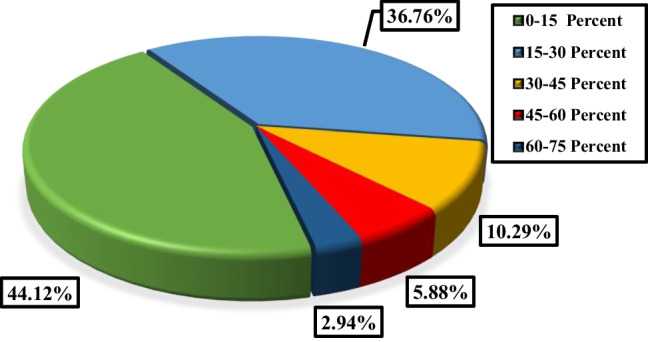
The percentage distribution of collectors’ thermal efficiency considering various ranges of the volume concentration of nanoparticles

In total, attaining better heat exchange rates is one of the original targets in industrial applications. By adding nanoparticles to the base fluid and creating a nanofluid, the conductivity of the working fluid may be increased. According to prior analyses, using nanoparticles and investigating the concentration, size, and types of nanoparticles have led to a noticeable improvement in thermal efficiency (Pandey and Chaurasiya 2017). Tang et al. (2010) investigated FPSCs with nanofluid aluminum oxide and copper oxide. They declared that utilizing nanofluid instead of water increased the collector's efficiency by 3.7%. Figure 13 shows the cent distribution of collector efficiency increase using nanofluid.

### Categorized outcomes of works concerning the FPSCs utilizing nanofluids

Researchers have turned to nanofluids as a promising way to improve the efficiency or performance of FPSCs. Numerous factors have been investigated in this study, which consists of the analysis of the type of nanoparticles employed, the type of nanofluid base fluid, the type of study using nanofluids, the volume concentration of employed nanofluids, and lastly, the thermal efficiency achieved through these advancements. The choice of base fluid is significant in the nanofluid formulation. Researchers have checked a wide range of base fluids, containing but not limited to water, engine oil, ethylene glycol, and even molten salts, to determine their impact on heat exchange efficiency. By changing the nanoparticles and the base fluid, researchers have sought to find the most effective compounds to increase the thermal performance of solar collectors. 

Several types of study using nanofluids have been included, spanning experimental, numerical, and theoretical analyses, which in this study is divided into two parts of experimental and non-experimental research. Each kind of research brings unique theories into the action of nanofluids in solar collectors, allowing for a comprehensive understanding of their heat exchange specifications and performance gains.

The volume concentration of the employed nanofluids significantly impacts the heat transfer efficiency. Researchers have investigated a wide range of volume concentrations to determine the collector’s thermal efficiency. Achieving the apropos equipoise is necessary, as excessively high concentrations may lead to particle aggregation, hindering the desirable enhancement.

Lastly, the thermal efficiency achieved through nanofluids in flat plate solar collectors is a significant parameter for appraising the success of this progress. Researchists have meticulously analyzed and measured thermal efficiency to assess the practical applicability of nanofluids in real-world solar collectors.

According to the studies conducted in the last 10 years regarding the selection of the type of nanoparticle, it can be said that the investigation of nanoparticles such as aluminum oxide and copper oxide alone does not justify innovation and great application. Another noteworthy point is the comprehensive investigation of these nanoparticles in different concentrations, which can be said to cover other further research to a large extent. On the other hand, according to the authors, due to its magnetic properties, hybrid nanoparticles, wall carbon nanotubes, and iron oxide can be a new approach for research and investigation of future researchers in this field.

Considering the increase in thermal efficiency of flat plate solar collectors along with the use of nanoparticles, it can be pointed out that the thermal efficiency increases with increasing concentration. Increasing concentration involves increasing the cost, increasing the pressure drop, severe sedimentation, etc., and that is why most of the research reviewed (as mentioned in “Volume concentration of employed nanofluids in FPSCs”) is in the range of 0–0.5.

Furthermore, as indicated by the research presented in this study, particularly in non-experimental analyses, there are instances where various outcomes have been reported. These discrepancies may stem from several sources, containing simulation errors and calculation, as well as potential inaccuracies in the measurement process.

## Employing inserts in FPSCs

As stated in the previous sections, new methods, including confusing agents, have been implemented to elevate heat exchange in various thermal systems. In the following, some research focusing on ways to ameliorate and optimize the heat exchange rate in flat plate solar collectors is given along with the insert.

**Fig. 14:**
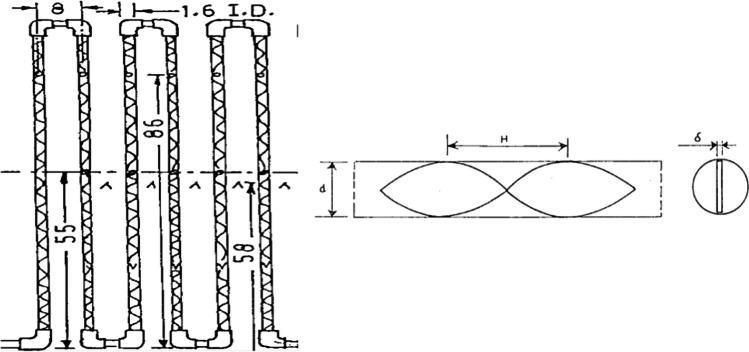
A flat plate solar collector with twisted tapes (Kumar and Prasad 2000)

In 2000, Kumar and Prasad (Kumar and Prasad 2000) tested a flat plate solar water heater implementing twisted tape inserts with various torsion ratios. The outcomes showed that the solar collector efficiency performed better than conventional samples despite the twisted tapes. According to the report, the performance improvement reached 30% (Fig. 14).

**Fig. 15:**
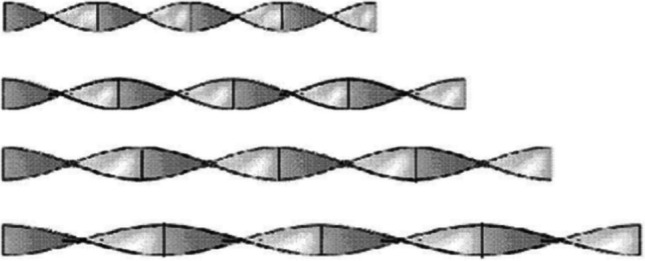
A view of the utilized twisted tape inserts with various lengths (Jaisankar et al. 2009)

In 2009, Jaisankar et al. (Jaisankar et al. 2009) assessed the presence of a twisted tape in a flat plate solar collector. Experimental data indicated that the presence of twisted tape went up the average Nusselt number and the coefficient of friction by 5.35 and 8.80%, respectively (Fig. 15).

**Fig. 16:**
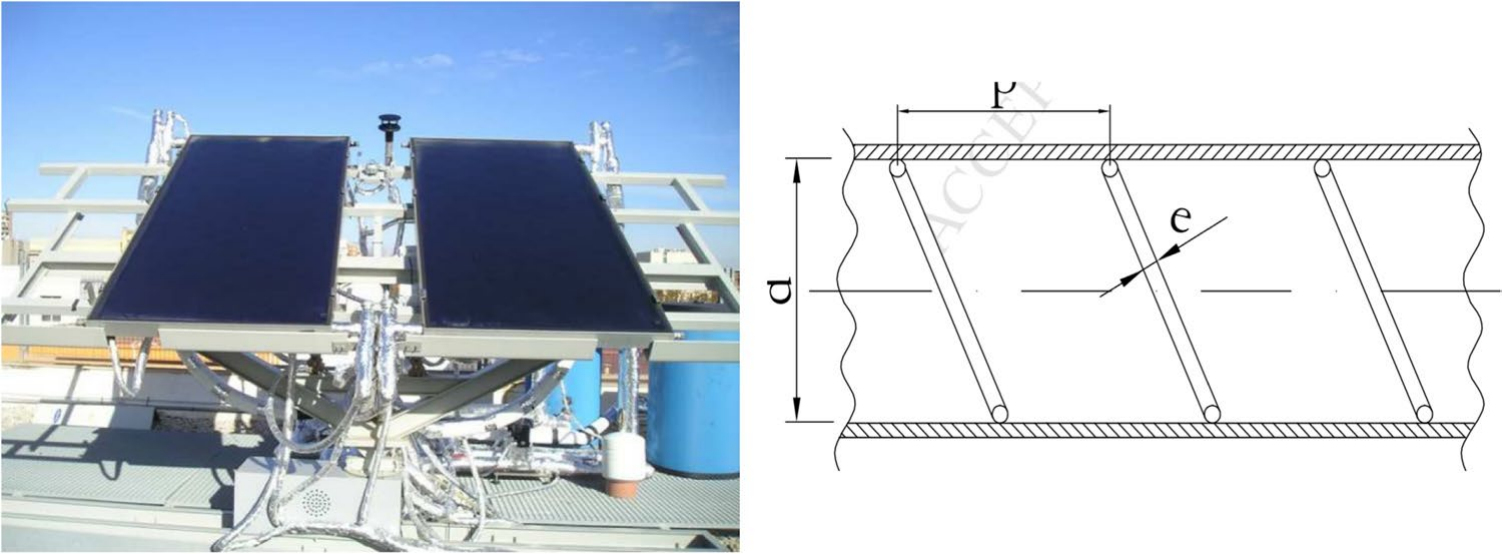
A view of a flat plate solar collector with wire coils (García et al. 2013)

Martin et al.) Martín et al. 2011 (checked the improvement of heat exchange in a flat panel solar collector by inserting wire coils with various working fluids. Conforming to the reported results, the collector’s thermal efficiency increased up to 4.5%. Garcia et al. (García et al. 2013) experimentally studied the heat transfer improvement in a flat plate solar water heater with coil insertion at five different mass flow rates. The outcomes illustrated that average thermal efficiency and useful power increased by 17% and 4%, respectively (Fig. 16).

**Fig. 17:**
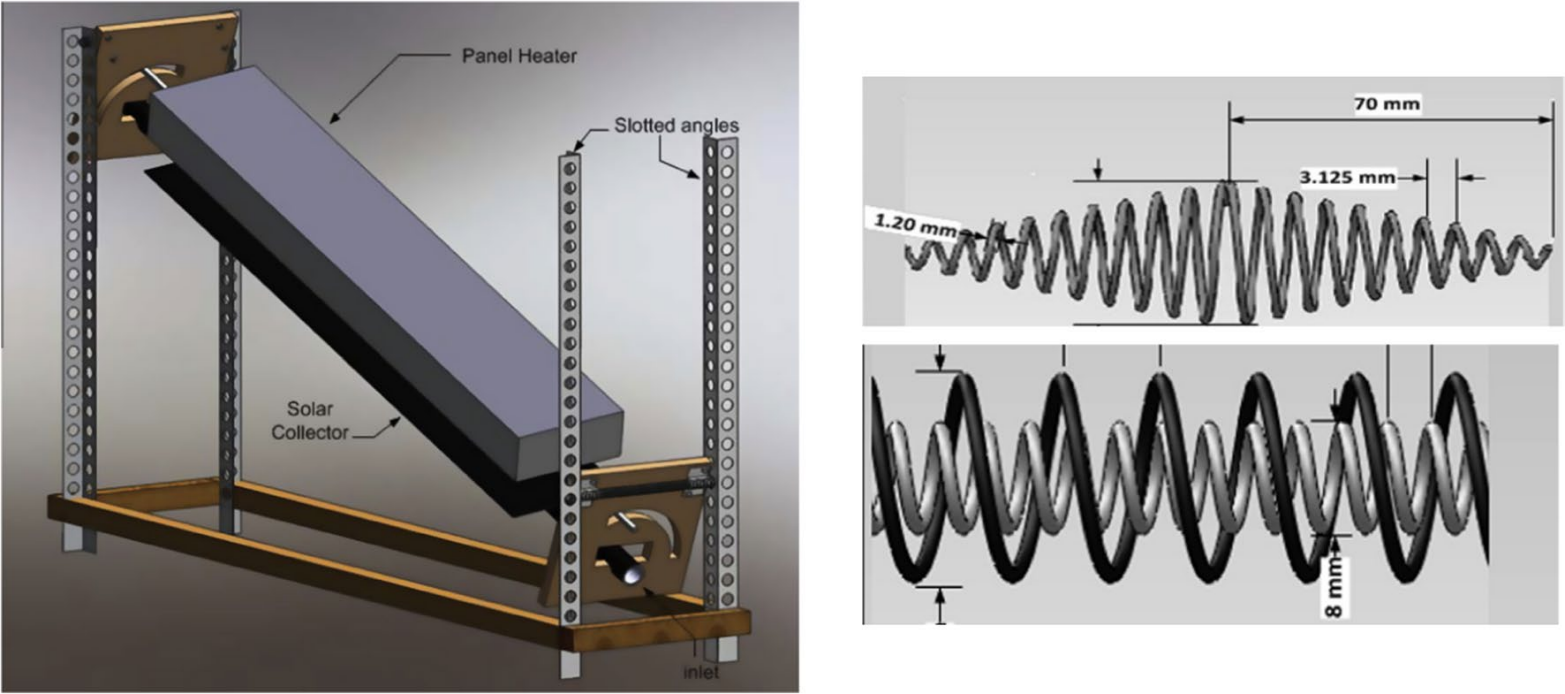
A view of a flat plate solar collector utilizing different types of inserts (Sandhu et al. 2014)

Using a twisted tape, Ananth and Jaisankar (Ananth and Jaisankar 2014) found that heat transfer got down with the rising distance between the strip and the length of the rod. Also, the heat transfer coefficient increased by 2.64 times when using twisted tape. Sandlow et al. (Sandhu et al. 2014) investigated the influence of a wire coil and a twisted tape on a flat plate collector. Based on the result, the maximum heat transfer coefficient was obtained for the concentric wire coils, so this increase even reached 460%. Some recent research focusing on solutions to ameliorate heat exchange rates by using inserts in flat plate collectors are listed in Fig. 17 and Table 2.

**Table 2:**
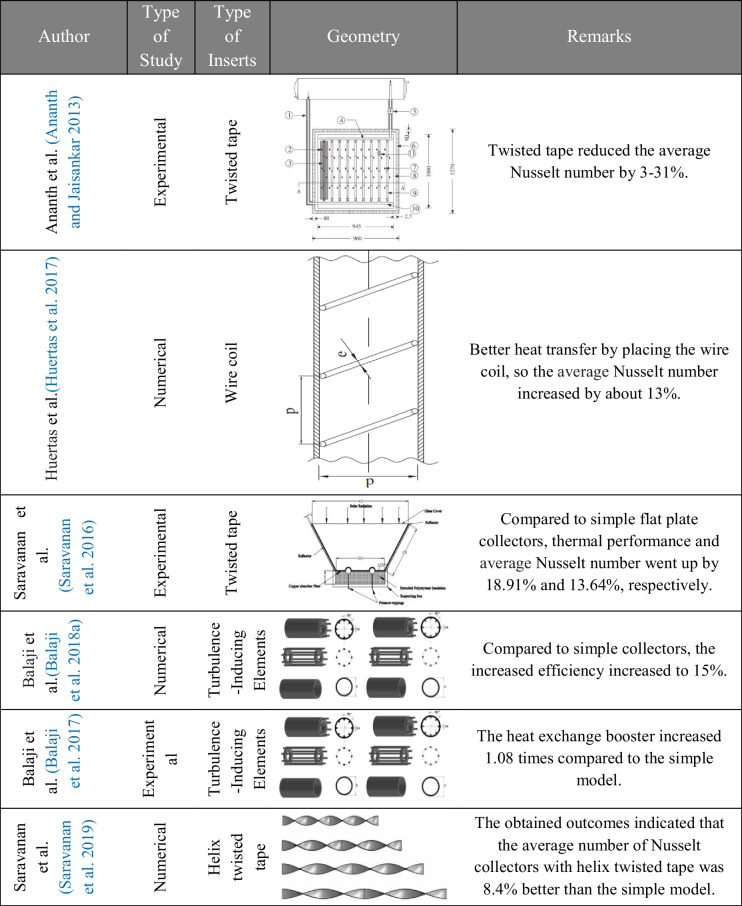

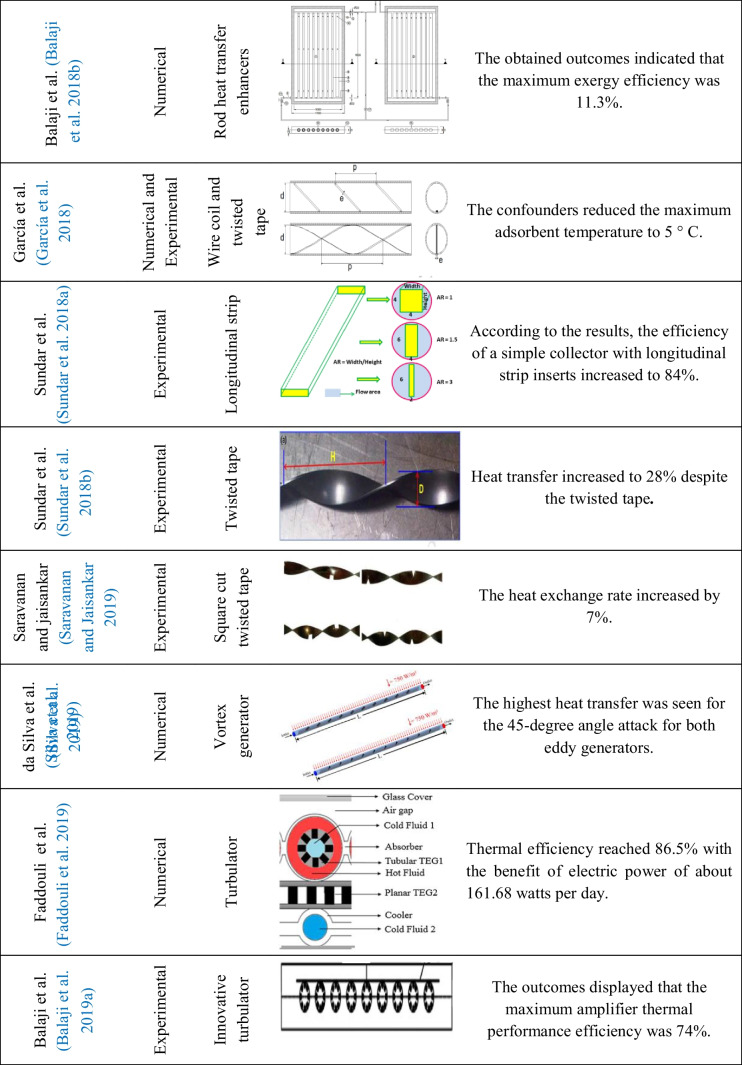

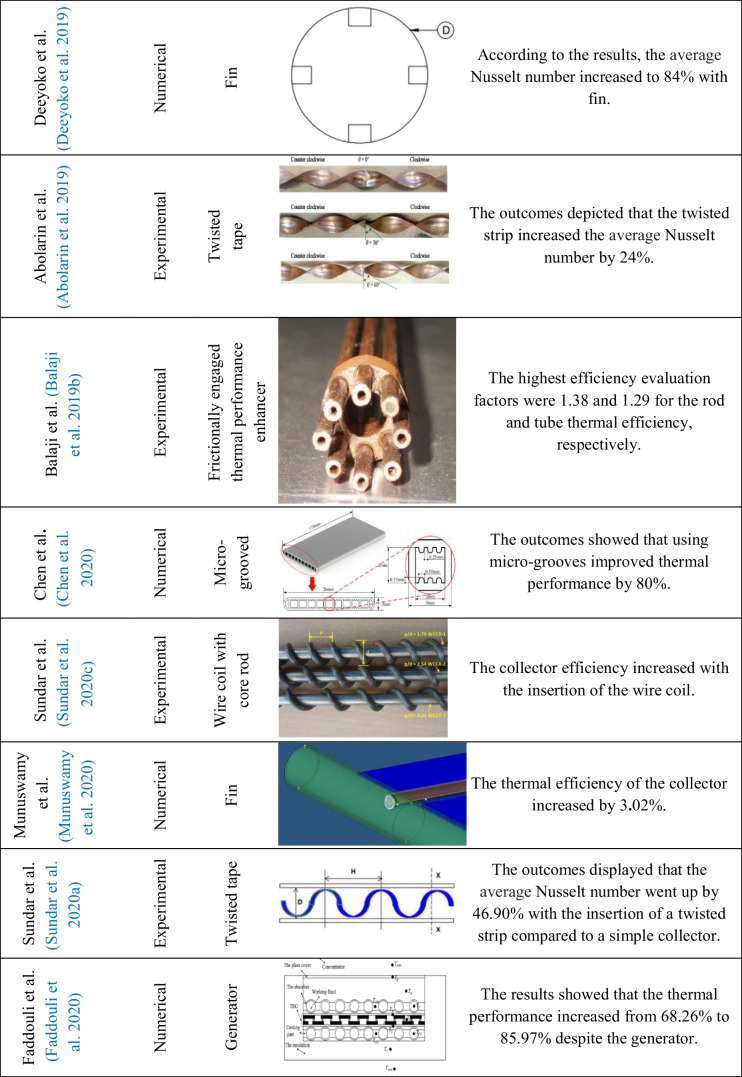

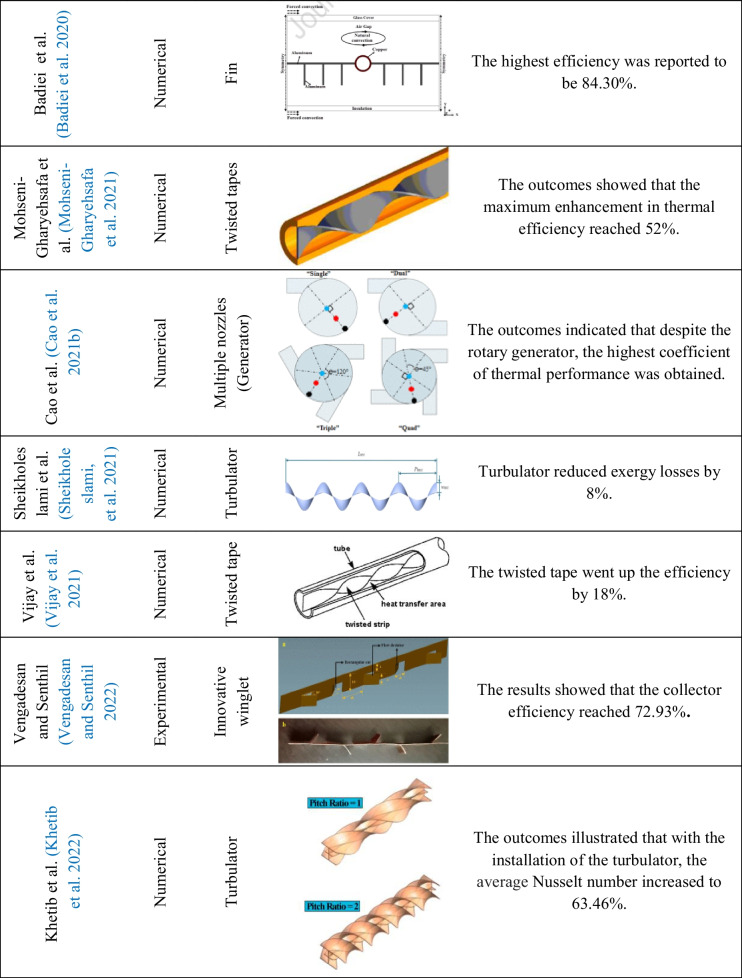

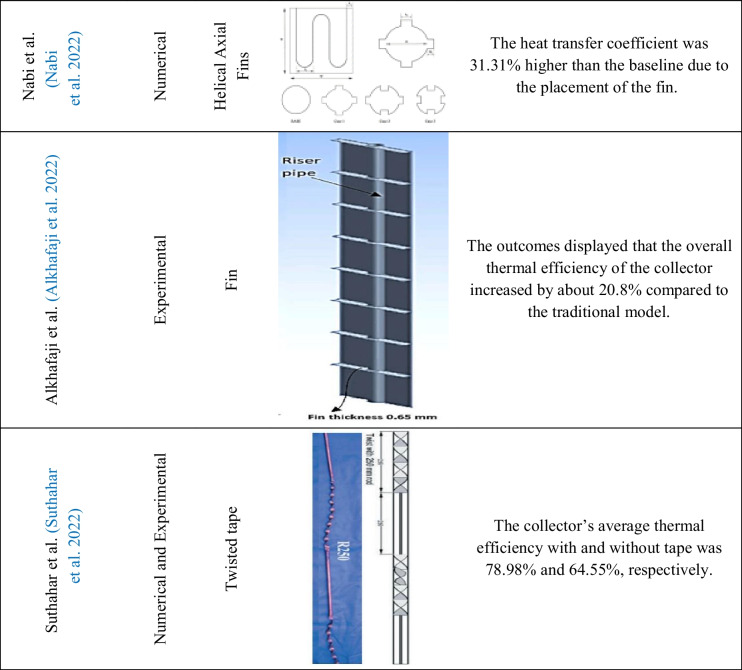
Overview of the studies of inserts used on flat plate solar collectors

**Fig. 18:**
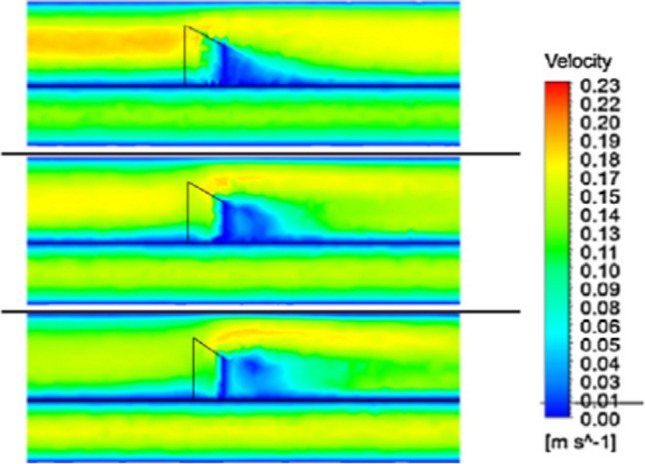
The velocity contour with winglet (da Silva et al. 2019)

According to Fig. 18, a low-velocity area immediately behind each winglet can be seen for winglet turbulators, known as fluid recirculation areas. These recirculation areas are more drastic for the higher angles of attack. Moreover, these zones are also seen for their insignificant heat exchange rate regarding low velocity and the slight temperature difference between the walls and fluid. Due to the plane’s situation in the tube, the primary target is to go up the mass flow on the winglet to produce more powerful longitudinal vortices.

**Fig. 19:**
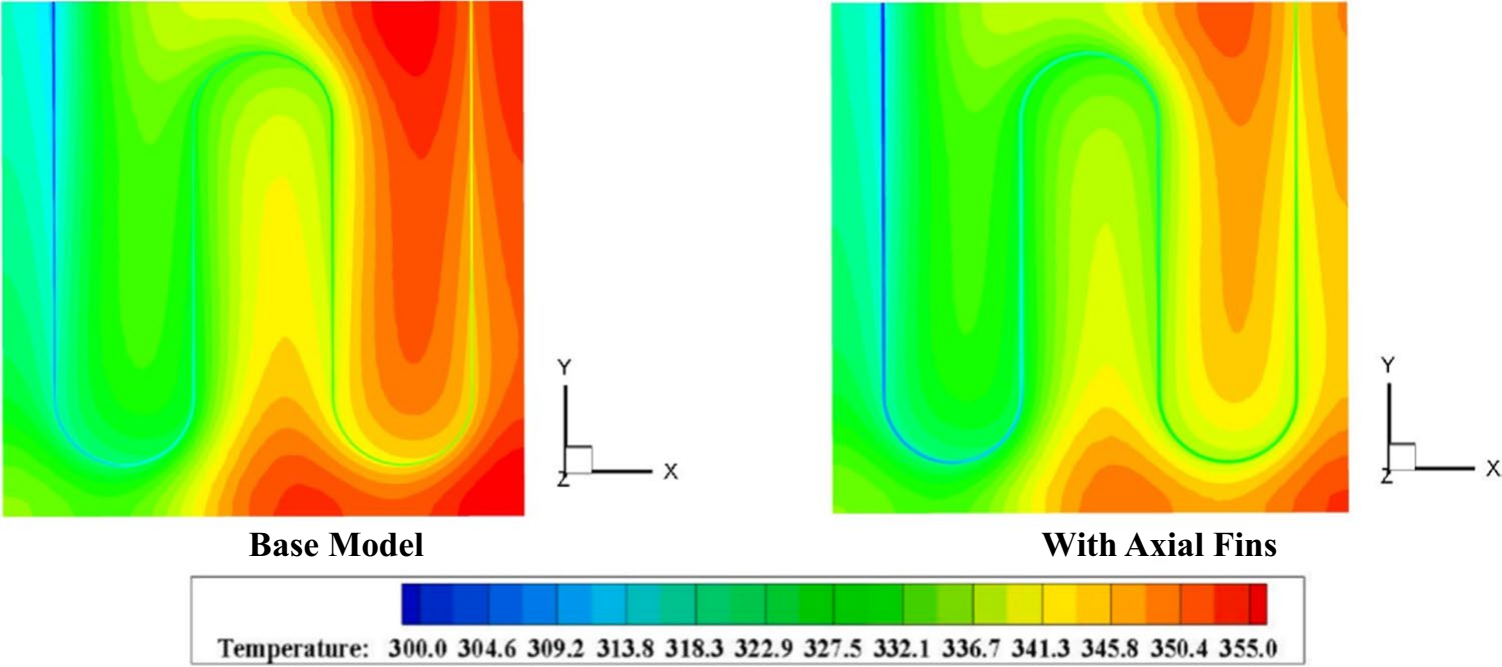
The temperature contour with fins (Nabi et al. 2022)

Figure 19 illustrates how the temperature is distributed in a flat plate solar collector. As you can see, the axial fins cause more circulation of the flows inside the collector. For this reason, the average temperature distribution of the collector is higher in the base model.

### Type of study in the field of FPSCs using inserts

**Fig. 20:**
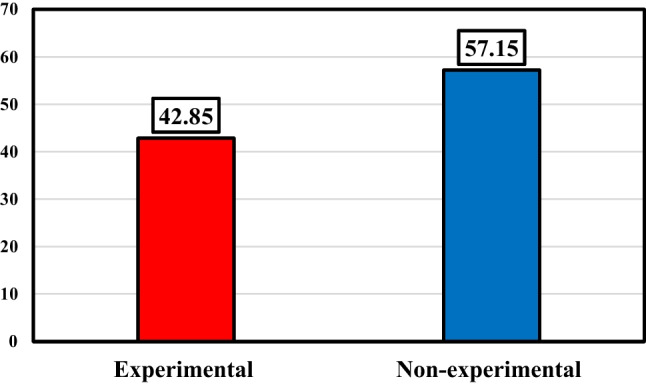
Usage breakdown of inserts on a flat plate collector

Conforming to Fig. 20, the considered studies are divided into two parts, experimental and non-experimental, in which the share of experimental research is about 42%, which is less compared to non-experimental studies. According to these outcomes, it can be said that conducting experimental studies has been given less attention than non-experimental studies due to the complexity of making all kinds of inserts and also the higher cost.

### Type of the employed inserts in FPSCs

**Fig. 21:**
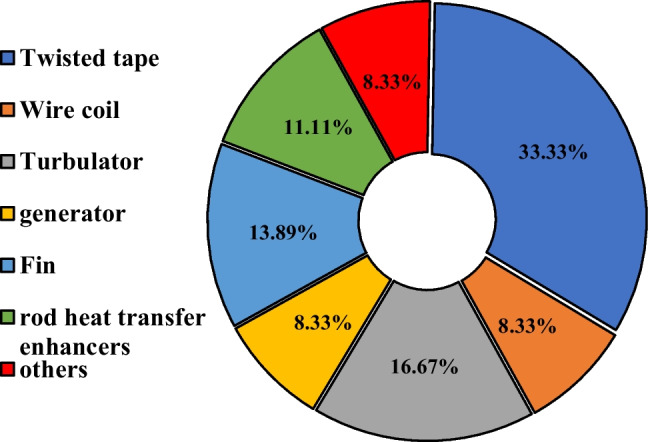
Breakdown of the use of various inserts on a flat plate collector

Based on the investigation, it can be seen from Fig. 21 that twisted tapes and turbulator had a significant contribution in past studies, with 33% and 16%, respectively. Also, fin inserts are attractive to researchers, with a share of 14% in the reviewed study. These observations may be attributed to these items’ simple manufacturing or lower cost than other inserts.

### Thermal efficiency of FPSCs equipped with inserts

**Fig. 22:**
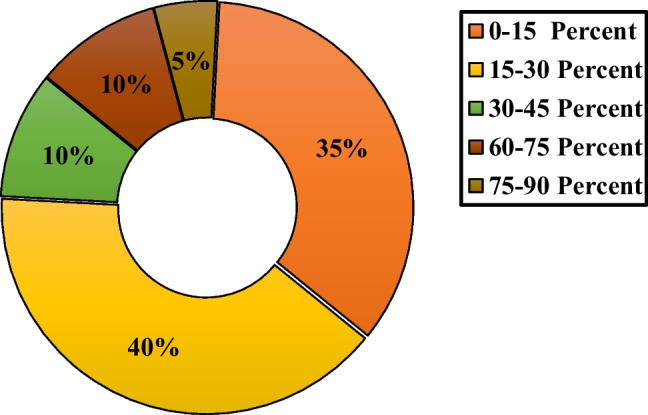
The percentage distribution of collector efficiency using inserts

Different studies to ameliorate the thermal efficiency of FPSCs have been conducted. This paper aims to assess the current two methods, namely, nanofluids and inserts (enhancement devices), to increase the thermal performance of FPSC. Inserts such as twisted tapes, wire coils, and turbulator improve heat transfer by increasing turbulence and swirling flow and decreasing the thickness of the boundary layer. Garcia et al. (García et al. 2013) studied the thermal efficiency improvement with coil insertion. The outcomes depicted that the average thermal efficiency went up by 17%. Figure 22 shows the percentage distribution of collector efficiency increase implementing inserts.

### Categorized outcomes of works concerning the FPSCs equipped with inserts

This part of the study delves into a comprehensive investigation of the vital influence resulting from the implementation of several inserts in flat plate solar collectors (FPSCs). Inserts, strategically placed within the collectors, have illustrated promising potential to go up their thermal efficiency. In this study, several factors are essential to understanding the effectiveness of using inserts in FPSCs, containing the type of study, the variety of employed inserts, and the assessment of thermal efficiency. Using inserts in FPSCs represents an approach to ameliorating heat exchange and thermal efficiency. Researchists have conducted many studies in this domain, utilizing experimental and non-experimental methods. By exploring the findings of these studies, valuable insights into the benefits and limitations of incorporating inserts into FPSC can be gained.

A varied range of inserts has been used to improve the thermal efficiency of FPSCs. These inserts come in different configurations and materials, each presenting unique heat exchange and flow increase characteristics. Some usual insert examples consist of twisted tapes, fins, and vortex generators, among others. Furthermore, this part examines the synergistic impacts of combining various kinds of inserts within FPSCs. Researchers have investigated diverse combinations of inserts to realize how they interact. In conclusion, integrating inserts into flat plate solar collectors illustrates a great way to enhance thermal efficiency and improve energy conversion.

Over the past decade, a significant trend in solar collectors has been the growing adoption of inserts to enhance and improve heat transfer, even at the expense of increased pressure drop. As mentioned in “Type of the employed inserts in FPSCs,” twisted tape is the most popular insert for researchers due to its simplicity and well-established manufacturing processes.

In fact, the implementation of inserts introduces a transformative influence on the flow regime and structure within the flat plate solar collectors. Each unique insert geometry brings about a distinct alteration, offering various approaches and methodologies for researchers to check. The versatility and adaptability of inserts contribute to a wealth of possibilities, making the future of research in this field significantly more precise and promising. As researchers continue to delve into novel shapes and configurations of inserts, the potential for further advancements and breakthroughs in enhancing heat transfer within flat plate solar collectors becomes increasingly evident. Thus, pursuing new and varied insert designs holds the key to unlocking even more significant potential in this area of research.

## Compound

Sometimes two or more active and passive approaches are used to meliorate thermal performance, producing higher heat transfer rates than would be provided by either technique individually. In the following, some research is given on ways to ameliorate and optimize the heat exchange rate in flat plate solar collectors, including inserts and nanofluids.

Sandra et al. (Sundar et al. 2018a) in 2018, with a laboratory study of the flat plate collector using aluminum oxide and inserting a longitudinal strip, found that the simple collector’s efficiency using nanofluid and longitudinal strip went up by 58 and 84%, respectively. Also, in 2020, they scrutinized the effect of employing twisted tape with copper nanoparticles on the thermal efficiency of the flat plate collector, which conforms to the obtained outcomes; the average Nusselt number increased to 46.90% compared to the simple collector. Also, the solar collector’s efficiency with base fluid is 52%, and the nanofluid’s presence went up by 58% (Sundar et al. 2020b).

Khatib et al. (Khetib et al. 2022), by numerical and laboratory investigation by combined nanofluid with turbulator, found that with the installation of turbulator, the average Nusselt number increased up to 63.46%. In addition, with the presence of nanofluid, energy efficiency was obtained about 22.19%. Table 3 shows some of the recent research focusing on ways to ameliorate and optimize the heat transfer rate in flat plate solar collectors, along with the inclusion of insert and nanofluids.

**Table 3:** Overview of the studies of nanofluids and inserts used on flat plate solar collectors

Author	Year	Type of study	nanofluid	type of inserts	Remarks
Sundar et al. (Sundar et al. 2018a)	2018	Experimental	Al_2_O_3_	Longitudinal strip	Simple collector efficiency using nanofluid and longitudinal strip increased by 58% and 84%, respectively
Sundar et al. (Sundar et al. 2018b)	2018	Experimental	Al_2_O_3_	Twisted tape	The growth in heat transfer despite the twisted tape reached 28%. Also, despite the nanofluid, the thermal efficiency improved by 18%
Sundar et al. (Sundar et al. 2020b)	**2020**	Experimental	Cu	Twisted tape	The outcomes displayed that the heat transfer coefficient went up by 46.90% compared to the simple collector with the insertion of a twisted tape. Also, the efficiency with water was reported to be 52% and increased to 58% despite the nanofluid
Sundar et al. (Sundar et al. 2020a)	2020	Experimental	Al_2_O_3_	Wire coil with core rod	The collector level decreased to 39.33%, and the collector efficiency increased by 20% despite the nanofluid
Munuswamy et al. (Munuswamy et al. 2020)	2020	Numerical	Al_2_O_3_ and CuO	Fin	The thermal efficiency increased by 7.2% with nanofluid and fin
Khetib et al. (Khetib et al. 2022)	2022	Numerical	Hybrid Nanofluid (DWCNTs-TiO_2_)	Turbulator	According to the results, with the installation of the turbulator, the average Nusselt number increased to 63.46%
Nabi et al. (Nabi et al. 2022)	2022	Numerical	Hybrid nanofluids MWCNT and SWCNT-CuO	Helical axial fins	The heat transfer coefficient was 31.31% higher than the baseline due to the fin’s placement and increased by 8.79% despite the nanofluid
Suthahar et al. (Suthahar et al. 2022)	2022	Numerical and experimental	Hybrid nanofluid (DWCNTs-TiO_2_)	Twisted tape	The average thermal efficiency with and without tape was 78.98% and 64.55%, respectively. In addition, the average thermal collector’s efficiency with nanofluid reached 84.85%

In this section, the recent investigations aimed at improving the heat transfer rate in flat plate solar collectors by simultaneous use of nanofluids and inserts are presented. These cutting-edge techniques offer promising avenues to improve the overall performance and efficiency of flat plate solar collectors.

## Conclusions

This work reviews the findings of the previously published research on flat plate solar collectors, in which the working fluid is nanofluid with a specific volume concentration, and a turbulator inside the collector has been used to ameliorate heat exchange. The use of nanofluid, owing to its enhanced thermos-physical properties compared to the pure fluid, leads to an increment in heat transfer rate from the solar collector to the working fluid. The results have shown that the selection of the type of nanoparticle and its volume concentration in the nanofluid, has a noteworthy impact on the value of augmentation the heat transfer rate. Employing a turbulator (vortex generator) inside the flat plate solar collector causes the formation of swirling and secondary flows in the fluid flow. Then, the swirling flow increases heat transfer between the working fluid and the collector. The investigations have shown that different types of turbulators have been used to enhance heat transfer in the collector model, and positive results have also been announced, which shows the importance of using the desired method in improving the collector’s thermal performance. Moreover, the investigations carried out in this work have shown that in a number of articles, both methods of using nanofluid (single or hybrid) and turbulator have been used as passive methods to ameliorate heat exchange in the FPCSs, and the obtained results were promising in enhancing the thermal performance of the collector.

## Data Availability

The data that support the findings of this study are available from the corresponding author upon reasonable request.

## Nomenclature

*A*: Area [m^2^]
*C*_P_: Specific heat capacity, [kJ/(kg K)]
*k*: Thermal conductivity [W/(m K)]
*T*: Temperature [℃

## Greek and symbols

*μ*: Viscosity [kg/m s]
*ρ*: Density [kg/m^3^]
*φ*: Solid volume fraction

## Abbreviation

Al_2_O_3_: Aluminum oxide
CeO_2_: Cerium oxide
CF: Covalent functionalized
CNC: Crystal nano-cellulose
CuO: Copper oxide
ETSCs: Evacuated tube solar collectors
Fe_3_O_4_: Iron oxide
FPSCs: Flat plate solar collector
GNPs: Graphene nanoplatelets
h-BN: Hexagonal boron nitride
LFSCs: Linear Fresnel solar collector
MgO: Magnesium oxide
MWCNTs: Multi walled carbon nanotubes
PDC: Parabolic dish collector
PTSCs: Parabolic tube solar collector
SiO2: Silicon dioxide
SWCNTs: Single-walled carbon nanotubes
TiO_2_: Titanium dioxide
ZnO: Zinc oxide

## Dimensionless parameter

*Nu*: Average Nusselt number, $\mathrm{Nu}=\frac{\mathrm{hd}}{\mathrm{k}}\mathrm{Nu}=\frac{\mathrm{hd}}{\mathrm{k}}\mathrm{Nu}=\frac{\mathrm{hd}}{\mathrm{k}}$ [nd]
*Re*: Reynolds number, $\mathrm{Re}=\frac{\mathrm{Pud}}{\upmu }\mathrm{Re}=\frac{\mathrm{Pud}}{\upmu }\mathrm{Re}=\frac{\mathrm{Pud}}{\upmu }$ [nd

## Index

*nf*: Nanofluid
*HNF*: *Hybrid nanofluid*
*np*: Nanoparticle

## References

[CR1] Abolarin S (2019). Heat transfer and pressure drop characteristics of alternating clockwise and counter clockwise twisted tape inserts in the transitional flow regime. Int J Heat Mass Transf.

[CR2] Aghili Yegane SP, Kasaeian A (2020). Thermal performance assessment of a flat-plate solar collector considering porous media, hybrid nanofluid and magnetic field effects. J Therm Anal Calorim.

[CR3] Ahmadi A (2016). Analysis of utilizing Graphene nanoplatelets to enhance thermal performance of flat plate solar collectors. Energy Convers Manag.

[CR4] Ajarostaghi SSM (2022). Hydrogen preheating in a PEMFC system employing a heat exchanger equipped with an innovative turbulator. Int J Hydrogen Energy.

[CR5] Akram N (2019). An experimental investigation on the performance of a flat-plate solar collector using eco-friendly treated graphene nanoplatelets–water nanofluids. J Therm Anal Calorim.

[CR6] Akram N (2021). Experimental investigations of the performance of a flat-plate solar collector using carbon and metal oxides based nanofluids. Energy.

[CR7] Alawaji SH (2001). Evaluation of solar energy research and its applications in Saudi Arabia—20 years of experience. Renew Sustain Energy Rev.

[CR8] Alawi OA (2019). Thermal efficiency of a flat-plate solar collector filled with pentaethylene glycol-treated graphene nanoplatelets: an experimental analysis. Sol Energy.

[CR9] Alim MA (2013). Analyses of entropy generation and pressure drop for a conventional flat plate solar collector using different types of metal oxide nanofluids. Energy Build.

[CR10] Alimoradi A (2017). Numerical investigation of heat transfer intensification in shell and helically coiled finned tube heat exchangers and design optimization. Chem Eng Process.

[CR11] Alkhafaji MH (2022). Study the influence of adding fins to the plate of the solar collector on thermal performance under natural phenomena. Int Commun Heat Mass Transfer.

[CR12] Alklaibi A (2021). Experimental analysis of exergy efficiency and entropy generation of diamond/water nanofluids flow in a thermosyphon flat plate solar collector. Int Commun Heat Mass Transfer.

[CR13] Allouhi A, Amine MB (2021). Heat pipe flat plate solar collectors operating with nanofluids. Sol Energy Mater Sol Cells.

[CR14] Alshuraiaan B (2022). Numerical study on charging performance of multi-enclosed thermal storage: Multiple versus integrated thermal storage. Case Stud Therm Eng.

[CR15] Alshuraiaan B (2023). Numerical studys on passive paramerters of a fluid-solid interaction problem derived by natural convection in a circular enclosure. Alex Eng J.

[CR16] Alzahrani AK (2021). Hybrid nanofluid flow in a Darcy-Forchheimer permeable medium over a flat plate due to solar radiation. Case Stud Therm Eng.

[CR17] Ameri M, Eshaghi MS (2018). Exergy and thermal assessment of a novel system utilizing flat plate collector with the application of nanofluid in porous media at a constant magnetic field. Thermal Sci Eng Progr.

[CR18] Ananth J, Jaisankar S (2013). Experimental studies on heat transfer and friction factor characteristics of thermosyphon solar water heating system fitted with regularly spaced twisted tape with rod and spacer. Energy Convers Manag.

[CR19] Ananth J, Jaisankar S (2014). Investigation on heat transfer and friction factor characteristics of thermosiphon solar water heating system with left-right twist regularly spaced with rod and spacer. Energy.

[CR20] Anderson B (1977) Solar energy: fundamentals in building design. United States: N. p. https://www.osti.gov/biblio/6563828

[CR21] Asadi A (2022). Numerical analysis of turbulence-inducing elements with various geometries and utilization of hybrid nanoparticles in a double pipe heat exchanger. Alex Eng J.

[CR22] Ashour AF (2022). Numerical investigation on the thermal performance of a flat plate solar collector using ZnO & CuO water nanofluids under Egyptian weathering conditions. Energy.

[CR23] Asker M, Gadanya TS (2019). Performance assessment of flat plate solar collector using different nanofluids. Hittite J Sci Eng.

[CR24] Badiei Z (2020). Performance improvements in solar flat plate collectors by integrating with phase change materials and fins: a CFD modeling. Energy.

[CR25] Baghel K (2021). Heat transfer characteristics of free surface water jet impingement on a curved surface. Int J Heat Mass Transf.

[CR26] Bahiraei M, Gharagozloo K (2020). Experimental investigation of hydrothermal characteristics for flow within a circular tube equipped with twisted conical strip inserts under different alignments. J Taiwan Inst Chem Eng.

[CR27] Bahiraei M (2021). Irreversibility features of a shell-and-tube heat exchanger fitted with novel trapezoidal oblique baffles: application of a nanofluid with different particle shapes. Int Commun Heat Mass Transfer.

[CR28] Balaji K (2017). Experimental investigation on heat transfer and pumping power of forced circulation flat plate solar collector using heat transfer enhancer in absorber tube. Appl Therm Eng.

[CR29] Balaji K (2018). Thermal performance of solar water heater using velocity enhancer. Renew Energy.

[CR30] Balaji K (2018). Exergy, economic and environmental analysis of forced circulation flat plate solar collector using heat transfer enhancer in riser tube. J Clean Prod.

[CR31] Balaji K (2019). Experimental analysis on free convection effect using two different thermal performance enhancers in absorber tube of a forced circulation flat plate solar water heater. Sol Energy.

[CR32] Balaji K (2019). Experimental investigation on flat plate solar collector using frictionally engaged thermal performance enhancer in the absorber tube. Renew Energy.

[CR33] Bazdidi-Tehrani F (2018). Flow and heat transfer analysis of TiO2/water nanofluid in a ribbed flat-plate solar collector. Renew Energy.

[CR34] Bellos E, Tzivanidis C (2018). Performance analysis and optimization of an absorption chiller driven by nanofluid based solar flat plate collector. J Clean Prod.

[CR35] Beltagy H (2017). Theoretical and experimental performance analysis of a Fresnel type solar concentrator. Renew Energy.

[CR36] Bergles A et al (1983) Bibliography on augmentation of convective heat and mass transfer-II. United States: N. p. 10.2172/5028987

[CR37] Bergles A, et al (1991) Literature review of heat transfer enhancement technology for heat exchanges in gas-ϐired applications, Report GRI 91–0146. Gas Research Institute.

[CR38] Bezaatpour M, Rostamzadeh H (2021). Design and evaluation of flat plate solar collector equipped with nanofluid, rotary tube, and magnetic field inducer in a cold region. Renew Energy.

[CR39] Bezaatpour M, Rostamzadeh H (2021). Simultaneous energy storage enhancement and pressure drop reduction in flat plate solar collectors using rotary pipes with nanofluid. Energy Build.

[CR40] Bianco V (2018). Numerical analysis of the Al2O3-water nanofluid forced laminar convection in an asymmetric heated channel for application in flat plate PV/T collector. Renew Energy.

[CR41] Borhani S (2019). Investigation of phase change in a spiral-fin heat exchanger. Appl Math Model.

[CR42] Buongiorno J (2006). Convective transport in nanofluids. J Heat Transfer.

[CR43] Cao Y (2021). Entropic analysis of a double helical tube heat exchanger including circular depressions on both inner and outer tube. Case Stud Therm Eng.

[CR44] Cao Y (2021). Inducing swirl flow inside the pipes of flat-plate solar collector by using multiple nozzles for enhancing thermal performance. Renew Energy.

[CR45] Chen G (2020). Thermal performance enhancement of micro-grooved aluminum flat plate heat pipes applied in solar collectors. Renew Energy.

[CR46] Cherif H (2019). A receiver geometrical details effect on a solar parabolic dish collector performance. Energy Rep.

[CR47] Choi SU, Eastman JA (1995) Enhancing thermal conductivity of fluids with nanoparticles. United States: N. p. https://www.osti.gov/biblio/196525

[CR48] Choudhary S (2020). Influence of stable zinc oxide nanofluid on thermal characteristics of flat plate solar collector. Renew Energy.

[CR49] Choudhary S (2020). Investigation of the stability of MgO nanofluid and its effect on the thermal performance of flat plate solar collector. Renew Energy.

[CR50] Choudhary S (2021). Time-based analysis of stability and thermal efficiency of flat plate solar collector using iron oxide nanofluid. Appl Therm Eng.

[CR51] Colangelo G (2015). Experimental test of an innovative high concentration nanofluid solar collector. Appl Energy.

[CR52] da Silva FA (2019). Longitudinal vortex generator applied to heat transfer enhancement of a flat plate solar water heater. Appl Therm Eng.

[CR53] Darbari B, Rashidi S (2021). Thermal efficiency of flat plate thermosyphon solar water heater with nanofluids. J Taiwan Inst Chem Eng.

[CR54] Deeyoko LAJ (2019). Exergy, economics and pumping power analyses of flat plate solar water heater using thermal performance enhancer in absorber tube. Appl Therm Eng.

[CR55] Edalatpour M, Solano JP (2017). Thermal-hydraulic characteristics and exergy performance in tube-on-sheet flat plate solar collectors: effects of nanofluids and mixed convection. Int J Therm Sci.

[CR56] El-Said EM (2021). Effect of curved segmental baffle on a shell and tube heat exchanger thermohydraulic performance: numerical investigation. Int J Therm Sci.

[CR57] Eltaweel M, Abdel-Rehim AA (2019). Energy and exergy analysis of a thermosiphon and forced-circulation flat-plate solar collector using MWCNT/Water nanofluid. Case Stud Therm Eng.

[CR58] Eltaweel M (2021). A comparison between flat-plate and evacuated tube solar collectors in terms of energy and exergy analysis by using nanofluid. Appl Therm Eng.

[CR59] Fadaei M (2023). Conjugated non-Newtonian phase change process in a shell and tube heat exchanger: a parametric-geometric analysis. Appl Therm Eng.

[CR60] Faddouli A (2019). Feasibility and performance investigation of a new smart system integrating planar/tubular thermoelectric generators with solar flat plate collector. Energy Convers Manag.

[CR61] Faddouli A (2020). Numerical analysis and performance investigation of new hybrid system integrating concentrated solar flat plate collector with a thermoelectric generator system. Renew Energy.

[CR62] Farajzadeh E (2018). Experimental and numerical investigations on the effect of Al2O3/TiO2H2O nanofluids on thermal efficiency of the flat plate solar collector. Renew Energy.

[CR63] Farhana K (2021). Analysis of efficiency enhancement of flat plate solar collector using crystal nano-cellulose (CNC) nanofluids. Sustain Energy Technol Assess.

[CR64] Gad M (2021). "Effect of different nanofluids on performance analysis of flat plate solar collector. J Dispersion Sci Technol.

[CR65] Gangadevi R, Vinayagam B (2019). Experimental determination of thermal conductivity and viscosity of different nanofluids and its effect on a hybrid solar collector. J Therm Anal Calorim.

[CR66] García A (2013). Experimental study of heat transfer enhancement in a flat-plate solar water collector with wire-coil inserts. Appl Therm Eng.

[CR67] García A (2018). The role of insert devices on enhancing heat transfer in a flat-plate solar water collector. Appl Therm Eng.

[CR68] Genc AM (2018). Thermal performance of a nanofluid-based flat plate solar collector: a transient numerical study. Appl Therm Eng.

[CR69] Georgeson L (2017). The global green economy: a review of concepts, definitions, measurement methodologies and their interactions. Geo: Geogr Environ.

[CR70] Giwa S (2021). A review of magnetic field influence on natural convection heat transfer performance of nanofluids in square cavities. J Therm Anal Calorim.

[CR71] Gnanavel C (2020). Heat transfer enhancement through nano-fluids and twisted tape insert with rectangular cut on its rib in a double pipe heat exchanger. Mater Today: Proceedings.

[CR72] Goel AK, Singh SN (2021). Experimental performance evaluation of an impinging jet with fins type solar air heater. Environ Sci Pollut Res.

[CR73] Gong J-H (2021). Comparative study of heat transfer enhancement using different fins in semi-circular absorber tube for large-aperture trough solar concentrator. Renew Energy.

[CR74] Gupta S (2021). Comparative performance analysis of flat plate solar collectors with and without aluminium oxide-based nano-fluid. Mater Today: Proceedings.

[CR75] Hamida MBB, Hatami M (2021). Investigation of heated fins geometries on the heat transfer of a channel filled by hybrid nanofluids under the electric field. Case Stud Thermal Eng.

[CR76] Hawwash A (2018). Numerical investigation and experimental verification of performance enhancement of flat plate solar collector using nanofluids. Appl Therm Eng.

[CR77] He Q (2015). Experimental investigation on the efficiency of flat-plate solar collectors with nanofluids. Appl Therm Eng.

[CR78] He J (2022). Heat transfer enhancement of impingement cooling with corrugated target surface. Int J Therm Sci.

[CR79] Hu Q (2021). A numerical study of heat transfer enhancement by helically corrugated tubes in the intermediate heat exchanger of a very-high-temperature gas-cooled reactor. Nucl Eng Des.

[CR80] Huertas A (2017). Tube-side heat transfer enhancement in flat-plate liquid solar collectors with wire coil inserts. Exp Heat Transfer.

[CR81] Hussein OA (2020). Thermal performance enhancement of a flat plate solar collector using hybrid nanofluid. Sol Energy.

[CR82] Izadi M (2020). Effects of porous material on transient natural convection heat transfer of nano-fluids inside a triangular chamber. Chin J Chem Eng.

[CR83] Izadi M, Assad MEH (2021). Use of nanofluids in solar energy systems.

[CR84] Izadi M (2013). Numerical study of developed laminar mixed convection of Al2O3/water nanofluid in an annulus. Chem Eng Commun.

[CR85] Izadi M (2013). Study on thermal and hydrodynamic indexes of a nanofluid flow in a micro heat sink. Chall Nano Micro Scale Sci Technol.

[CR86] Izadi M (2015). Effects of inclination angle on mixed convection heat transfer of a nanofluid in a square cavity. Int J Comput Methods Eng Sci Mech.

[CR87] Izadi M (2015). Modeling of effective thermal conductivity and viscosity of carbon structured nanofluid. Chall Nano Micro Scale Sci Technol.

[CR88] Izadi M (2018). Numerical simulation of natural convection heat transfer inside a┴ shaped cavity filled by a MWCNT-Fe3O4/water hybrid nanofluids using LBM. Chem Eng Process-Process Intensification.

[CR89] Izadi M (2019). Natural convection of a magnetizable hybrid nanofluid inside a porous enclosure subjected to two variable magnetic fields. Int J Mech Sci.

[CR90] Izadi M (2020). Numerical simulation of thermogravitational energy transport of a hybrid nanoliquid within a porous triangular chamber using the two-phase mixture approach. Adv Powder Technol.

[CR91] Izadi M (2022). Analysis of applying fin for charging process of phase change material inside H-shaped thermal storage. Int Commun Heat Mass Transfer.

[CR92] Izadi M (2022). Charging process of a partially heated trapezoidal thermal energy storage filled by nano-enhanced PCM using controlable uniform magnetic field. Int Commun Heat Mass Transfer.

[CR93] Izadi M (2023). Numerical study on forced convection heat transfer of TiO2/water nanofluid flow inside a double-pipe heat exchanger with spindle-shaped turbulators. Eng Anal Boundary Elem.

[CR94] Izadi M (2023). Influence of finned charges on melting process performance in a thermal energy storage. Thermal Sci Eng Prog.

[CR95] Izadi M, et al (2023c) Transient magneto-buoyant convection of a magnetizable nanofluid inside a circle sensible storage subjected to double time-dependent thermal sources. J Therm Anal Calorimet 1–21

[CR96] Jaisankar S (2009). Experimental studies on heat transfer and friction factor characteristics of forced circulation solar water heater system fitted with helical twisted tapes. Sol Energy.

[CR97] Jalali E (2019). Heat transfer of oil/MWCNT nanofluid jet injection inside a rectangular microchannel. Symmetry.

[CR98] Jamal-Abad MT (2017). Experimental investigation on a solar parabolic trough collector for absorber tube filled with porous media. Renew Energy.

[CR99] Javadi H (2021). Impact of employing hybrid nanofluids as heat carrier fluid on the thermal performance of a borehole heat exchanger. Energies.

[CR100] Jouybari HJ (2017). Effects of porous material and nanoparticles on the thermal performance of a flat plate solar collector: an experimental study. Renew Energy.

[CR101] Kalogirou S (2004) Solar thermal collectors and applications progress in energy and combustion science, edition: London. Washington DC. 10.1016/j.pecs.2004.02.001

[CR102] Kang W (2017). Economic analysis of flat-plate and U-tube solar collectors using an Al2O3 nanofluid. Energies.

[CR103] Kashyap Y, et al (2018) Exergy analysis of a flat plate solar collector with grooved absorber tube configuration using aqueous ZnO–ethylene glycol. J Solar Energy Eng 140(6):061011. 10.1115/1.4040582

[CR104] Kazaz O, et al (2022) Numerical investigation of the influences of nanoparticle size and tilt angle in a directly absorption solar system. https://eprints.gla.ac.uk/273438/#

[CR105] Khetib Y (2022). Influence of using innovative turbulators on the exergy and energy efficacy of flat plate solar collector with DWCNTs-TiO2/water nanofluid. Sustain Energy Technol Assess.

[CR106] Kiliç F (2018). Effect of titanium dioxide/water nanofluid use on thermal performance of the flat plate solar collector. Sol Energy.

[CR107] Kim H (2017). Experimental study on performance improvement of U-tube solar collector depending on nanoparticle size and concentration of Al2O3 nanofluid. Energy.

[CR108] Kreith F, Kreider JF (1978) Principles of solar engineering. United States: N. p. https://www.osti.gov/biblio/6114590

[CR109] Kumar A, Prasad B (2000). Investigation of twisted tape inserted solar water heaters—heat transfer, friction factor and thermal performance results. Renew Energy.

[CR110] Kumar A (2021). An up-to-date review on evacuated tube solar collectors. J Therm Anal Calorim.

[CR111] Kumar LH (2021). Energy, exergy and economic analysis of liquid flat-plate solar collector using green covalent functionalized graphene nanoplatelets. Appl Therm Eng.

[CR112] Kumar LH (2022). Experimental study on the effect of bio-functionalized graphene nanoplatelets on the thermal performance of liquid flat plate solar collector. J Therm Anal Calorim.

[CR113] Lanjwani H (2021). Dual solutions of time-dependent magnetohydrodynamic stagnation point boundary layer micropolar nanofluid flow over shrinking/stretching surface. Appl Math Mech.

[CR114] Léal L (2013). An overview of heat transfer enhancement methods and new perspectives: focus on active methods using electroactive materials. Int J Heat Mass Transf.

[CR115] Lee M (2020). Performance evaluation of flat plate and vacuum tube solar collectors by applying a MWCNT/Fe3O4 binary nanofluid. Energies.

[CR116] Li Z (2020). Numerical assessment on the hydrothermal behavior and irreversibility of MgO-Ag/water hybrid nanofluid flow through a sinusoidal hairpin heat-exchanger. Int Commun Heat Mass Transfer.

[CR117] Liu P (2018). An experimental and numerical study on the laminar heat transfer and flow characteristics of a circular tube fitted with multiple conical strips inserts. Int J Heat Mass Transf.

[CR118] Lovegrove K (2011). A new 500 m2 paraboloidal dish solar concentrator. Sol Energy.

[CR119] Mahamude ASF (2022). Experimental study on the efficiency improvement of flat plate solar collectors using hybrid nanofluids graphene/waste cotton. Energies.

[CR120] Mamori H (2021). Heat transfer enhancement and torque reduction by traveling wave-like blowing and suction in turbulent Taylor-Couette flow. J Therm Sci Technol.

[CR121] Martín RH (2011). Simulation of an enhanced flat-plate solar liquid collector with wire-coil insert devices. Sol Energy.

[CR122] Mashayekhi R (2022). Heat transfer enhancement of nanofluid flow in a tube equipped with rotating twisted tape inserts: a two-phase approach. Heat Transfer Eng.

[CR123] Mehryan S (2020). Numerical study on natural convection of Ag–MgO hybrid/water nanofluid inside a porous enclosure: a local thermal non-equilibrium model. Powder Technol.

[CR124] Mehryan S (2020). Free convection in a trapezoidal enclosure divided by a flexible partition. Int J Heat Mass Transf.

[CR125] Meibodi SS (2015). Experimental investigation on the thermal efficiency and performance characteristics of a flat plate solar collector using SiO2/EG–water nanofluids. Int Commun Heat Mass Transfer.

[CR126] Meibodi SS (2016). Second law analysis of a nanofluid-based solar collector using experimental data. J Therm Anal Calorim.

[CR127] Michael JJ, Iniyan S (2015). Performance of copper oxide/water nanofluid in a flat plate solar water heater under natural and forced circulations. Energy Convers Manag.

[CR128] Mirzaei M (2018). Assessment of Al2O3 nanoparticles for the optimal operation of the flat plate solar collector. Appl Therm Eng.

[CR129] Modi AJ (2020). Thermal performance augmentation of fin-and-tube heat exchanger using rectangular winglet vortex generators having circular punched holes. Int J Heat Mass Transf.

[CR130] Mohammadpour J (2022). Machine learning regression-CFD models for the nanofluid heat transfer of a microchannel heat sink with double synthetic jets. Int Commun Heat Mass Transfer.

[CR131] Mohseni-Gharyehsafa B (2021). Soft computing analysis of thermohydraulic enhancement using twisted tapes in a flat-plate solar collector: sensitivity analysis and multi-objective optimization. J Clean Prod.

[CR132] Moravej M (2020). Enhancing the efficiency of a symmetric flat-plate solar collector via the use of rutile TiO2-water nanofluids. Sustain Energy Technol Assess.

[CR133] Motahhir S (2019). Open hardware/software test bench for solar tracker with virtual instrumentation. Sustain Energy Technol Assess.

[CR134] Mousavi Ajarostaghi SS (2022). A review of recent passive heat transfer enhancement methods. Energies.

[CR135] Munuswamy DB (2020). Experimental investigation on lowering the environmental hazards and improving the performance patterns of solar flat plate collectors by employing the internal longitudinal fins and nano additives. Environ Sci Pollut Res.

[CR136] Mustafa J (2021). Challenging of using CuO nanoparticles in a flat plate solar collector-energy saving in a solar-assisted hot process stream. J Taiwan Inst Chem Eng.

[CR137] Nabi H (2022). Increasing heat transfer in flat plate solar collectors using various forms of turbulence-inducing elements and CNTs-CuO hybrid nanofluids. Case Stud Therm Eng.

[CR138] Nawsud ZA (2022). A comprehensive review on the use of nano-fluids and nano-PCM in parabolic trough solar collectors (PTC). Sustain Energy Technol Assess.

[CR139] Nazir MS (2021). A comprehensive review of parabolic trough solar collectors equipped with turbulators and numerical evaluation of hydrothermal performance of a novel model. Sustain Energy Technol Assess.

[CR140] Noorbakhsh M (2022). Thermal analysis of nanofluids flow in a double pipe heat exchanger with twisted tapes insert in both sides. J Therm Anal Calorim.

[CR141] Okonkwo EC (2020). Thermodynamic evaluation and optimization of a flat plate collector operating with alumina and iron mono and hybrid nanofluids. Sustain Energy Technol Assess.

[CR142] Pal RK, Kumar R (2021). Investigations of thermo-hydrodynamics, structural stability, and thermal energy storage for direct steam generation in parabolic trough solar collector: a comprehensive review. J Clean Prod.

[CR143] Pandey KM, Chaurasiya R (2017). A review on analysis and development of solar flat plate collector. Renew Sustain Energy Rev.

[CR144] Papadimitratos A (2016). Evacuated tube solar collectors integrated with phase change materials. Sol Energy.

[CR145] Peng G (2021). Potential and challenges of improving solar still by micro/nano-particles and porous materials-a review. J Clean Prod.

[CR146] Purohit N (2018). Heat transfer and entropy generation analysis of alumina/water nanofluid in a flat plate PV/T collector under equal pumping power comparison criterion. Renew Energy.

[CR147] Rajput NS (2019). Performance analysis of flat plate solar collector using Al2O3/distilled water nanofluid: an experimental investigation. Mater Today: Proceedings.

[CR148] Rashidi S (2019). Combination of nanofluid and inserts for heat transfer enhancement. J Therm Anal Calorim.

[CR149] Rashidi S (2021). Progress and challenges of helical-shaped geothermal heat exchangers. Environ Sci Pollut Res.

[CR150] Rungasamy A (2021). A review of linear Fresnel primary optical design methodologies. Sol Energy.

[CR151] Sabiha M (2015). Energy performance of an evacuated tube solar collector using single walled carbon nanotubes nanofluids. Energy Convers Manag.

[CR152] Saedodin S (2020). Effect of twisted turbulator and various metal oxide nanofluids on the thermal performance of a straight tube: numerical study based on experimental data. Chem Eng Process-Process Intensification.

[CR153] Saedodin S (2021). Hydrothermal analysis of heat transfer and thermal performance characteristics in a parabolic trough solar collector with turbulence-Inducing elements. Sustain Energy Technol Assess.

[CR154] Saedodin S (2023). Statistical analysis and shape optimization of a finned corrugated heat exchanger using RSM. Chem Eng Commun.

[CR155] Saffarian MR (2020). Heat transfer enhancement in a flat plate solar collector with different flow path shapes using nanofluid. Renew Energy.

[CR156] Said Z (2014). Analyses of exergy efficiency and pumping power for a conventional flat plate solar collector using SWCNTs based nanofluid. Energy Build.

[CR157] Said Z (2015). Performance enhancement of a flat plate solar collector using titanium dioxide nanofluid and polyethylene glycol dispersant. J Clean Prod.

[CR158] Said Z (2015). Thermophysical properties of single wall carbon nanotubes and its effect on exergy efficiency of a flat plate solar collector. Sol Energy.

[CR159] Said Z (2016). "Energy and exergy analysis of a flat plate solar collector using different sizes of aluminium oxide based nanofluid. J Clean Prod.

[CR160] Said Z (2016). Energy and exergy efficiency of a flat plate solar collector using pH treated Al2O3 nanofluid. J Clean Prod.

[CR161] Sajjadi H (2021). Natural convection heat transfer in a porous cavity with sinusoidal temperature distribution using Cu/water nanofluid: Double MRT lattice Boltzmann method. Commun Comput Phys.

[CR162] Saleh B, et al (2022) The combined effect of Al2O3 nanofluid and coiled wire inserts in a flat-plate solar collector on heat transfer, thermal efficiency and environmental CO2 characteristics. Arabian J Sci Eng 1–28.

[CR163] Sandhu G (2014). Experimental study on the combined effects of inclination angle and insert devices on the performance of a flat-plate solar collector. Int J Heat Mass Transf.

[CR164] Saravanan A, Jaisankar S (2019). Heat transfer augmentation techniques in forced flow V-trough solar collector equipped with V-cut and square cut twisted tape. Int J Therm Sci.

[CR165] Saravanan A (2016). Experimental studies on heat transfer and friction factor characteristics of twist inserted V-trough thermosyphon solar water heating system. Energy.

[CR166] Saravanan A (2019). Influence of helix twisted tape on heat transfer and friction factor in forced circulation V-trough solar water heater. Int J Sustain Energ.

[CR167] Sarsam WS (2020). Thermal performance of a flat-plate solar collector using aqueous colloidal dispersions of graphene nanoplatelets with different specific surface areas. Appl Therm Eng.

[CR168] Saydam V (2019). Design and experimental analysis of a helical coil phase change heat exchanger for thermal energy storage. J Energy Storage.

[CR169] Shamshirgaran SR (2018). Upper limits for the work extraction by nanofluid-filled selective flat-plate solar collectors. Energy.

[CR170] Shang Y (2022). The computational study of microchannel thickness effects on H2O/CuO nanofluid flow with molecular dynamics simulations. J Mol Liq.

[CR171] Sharafeldin M, Gróf G (2018). Experimental investigation of flat plate solar collector using CeO2-water nanofluid. Energy Convers Manag.

[CR172] Sharafeldin MA (2017). Experimental study on the performance of a flat-plate collector using WO3/Water nanofluids. Energy.

[CR173] Sharma, S., et al. (2020). Thermal performance enhancement of flat-plate solar collector using CeO 2–water nanofluid. Advances in Solar Power Generation and Energy Harvesting, Springer**,** 109–118.

[CR174] Shehzad S (2021). Influence of fin orientation on the natural convection of aqueous-based nano-encapsulated PCMs in a heat exchanger equipped with wing-like fins. Chem Eng Process-Process Intensification.

[CR175] Shehzad S (2021). Rheological features of non-Newtonian nanofluids flows induced by stretchable rotating disk. Phys Scr.

[CR176] Sheikholeslami M (2021). Performance of solar collector with turbulator involving nanomaterial turbulent regime. Renew Energy.

[CR177] Shojaeizadeh E (2015). Exergy efficiency investigation and optimization of an Al2O3–water nanofluid based flat-plate solar collector. Energy Build.

[CR178] Sint NKC (2017). Theoretical analysis to determine the efficiency of a CuO-water nanofluid based-flat plate solar collector for domestic solar water heating system in Myanmar. Sol Energy.

[CR179] Stalin MJP (2019). Experimental and theoretical investigation on the effects of lower concentration CeO2/water nanofluid in flat-plate solar collector. J Therm Anal Calorim.

[CR180] Stalin MJP (2020). Energy, economic and environmental investigation of a flat plate solar collector with CeO2/water nanofluid. J Therm Anal Calorim.

[CR181] Sundar LS (2018). Effectiveness analysis of solar flat plate collector with Al2O3 water nanofluids and with longitudinal strip inserts. Int J Heat Mass Transf.

[CR182] Sundar LS (2018). Experimental investigation of Al2O3/water nanofluids on the effectiveness of solar flat-plate collectors with and without twisted tape inserts. Renew Energy.

[CR183] Sundar LS (2020). Efficiency, energy and economic analysis of twisted tape inserts in a thermosyphon solar flat plate collector with Cu nanofluids. Renew Energy Focus.

[CR184] Sundar LS (2020). Properties, heat transfer, energy efficiency and environmental emissions analysis of flat plate solar collector using nanodiamond nanofluids. Diam Relat Mater.

[CR185] Sundar LS (2020). Energy, efficiency, economic impact, and heat transfer aspects of solar flat plate collector with Al2O3 nanofluids and wire coil with core rod inserts. Sustain Energy Technol Assess.

[CR186] Sundar LS (2021). Solar energy absorbed thermosyphon flat plate collector analysis using Cu/H2O nanofluid–an experimental study. Energy Clim Chang.

[CR187] Suthahar SJ (2022). Experimental investigation on solar flat plate collector using alumina nanofluid with tube inserts. Mater Technol.

[CR188] Tahat MS, Benim AC (2017) Experimental analysis on thermophysical properties of Al2O3/CuO hybrid nano fluid with its effects on flat plate solar collector. Defect and diffusion forum, Trans Tech Publ. 10.4028/www.scientific.net/DDF.374.148

[CR189] Tang R (2010). Experimental and modeling studies on thermosiphon domestic solar water heaters with flat-plate collectors at clear nights. Energy Convers Manag.

[CR190] Taylor R (2013). Small particles, big impacts: a review of the diverse applications of nanofluids. J Appl Phys.

[CR191] Tong Y (2019). Energy and exergy comparison of a flat-plate solar collector using water, Al2O3 nanofluid, and CuO nanofluid. Appl Therm Eng.

[CR192] Uosofvand H, Abbasian Arani AA (2021). Shell-and-tube heat exchangers performance improvement employing hybrid segmental–helical baffles and ribbed tubes combination. J Braz Soc Mech Sci Eng.

[CR193] Valipour P (2017). Influence of magnetic field on CNT-polyethylene nanofluid flow over a permeable cylinder. J Mol Liq.

[CR194] Valipour P (2018). Two phase model for nanofluid heat transfer intensification in a rotating system under the effect of magnetic field. Chem Eng Process-Process Intensification.

[CR195] Vengadesan E, Senthil R (2022). Experimental study on the thermal performance of a flat plate solar water collector with a bifunctional flow insert. Sustain Energy Technol Assess.

[CR196] Verma SK (2016). Performance augmentation in flat plate solar collector using MgO/water nanofluid. Energy Convers Manage.

[CR197] Verma SK (2017). Experimental evaluation of flat plate solar collector using nanofluids. Energy Convers Manag.

[CR198] Verma SK (2018). Performance analysis of hybrid nanofluids in flat plate solar collector as an advanced working fluid. Sol Energy.

[CR199] Vijay R (2021). Performance study of FPSC integrated with twisted tape inserts. Mater Today: Proceedings.

[CR200] Vincely DA, Natarajan E (2016). Experimental investigation of the solar FPC performance using graphene oxide nanofluid under forced circulation. Energy Convers Manag.

[CR201] Weiss W, Mauthner F (2010) Solar heat worldwide. Markets and contribution to the energy supply. 10.18777/ieashc-shw-2022-0001

[CR202] Xiong Q (2021). A comprehensive review on the application of hybrid nanofluids in solar energy collectors. Sustain Energy Technol Assess.

[CR203] Xiong Q (2021). State-of-the-art review of nanofluids in solar collectors: a review based on the type of the dispersed nanoparticles. J Clean Prod.

[CR204] Xiong Q (2021). Numerical analysis of porous flat plate solar collector under thermal radiation and hybrid nanoparticles using two-phase model. Sustain Energy Technol Assess.

[CR205] Xiong Q (2022). Application of phase change material in improving trombe wall efficiency: an up-to-date and comprehensive overview. Energy Build.

[CR206] Xu F (2022). Influence of key geometry parameters on heat transfer and flow resistance properties of toothed internal spiral piece cracking furnace tube. Appl Therm Eng.

[CR207] Yang C (2021). Heat transfer study of a hybrid smooth and spirally corrugated tube. Heat Transfer Eng.

[CR208] Yousefi T (2012). An experimental investigation on the effect of Al2O3–H2O nanofluid on the efficiency of flat-plate solar collectors. Renew Energy.

[CR209] Zaboli M (2019). Effects of geometrical and operational parameters on heat transfer and fluid flow of three various water based nanofluids in a shell and coil tube heat exchanger. SN Appl Sci.

[CR210] Zhai C (2019). Heat transfer augmentation in a circular tube with delta winglet vortex generator pairs. Int J Therm Sci.

[CR211] Zheng L (2017). Numerical investigation on heat transfer performance and flow characteristics in circular tubes with dimpled twisted tapes using Al2O3-water nanofluid. Int J Heat Mass Transf.

[CR212] Zheng X (2018). Numerical investigation on paraffin/expanded graphite composite phase change material based latent thermal energy storage system with double spiral coil tube. Appl Therm Eng.

[CR213] Zhou W (2022). Effects of mechanical vibration on the heat transfer performance of shell-and-tube latent heat thermal storage units during charging process. Appl Therm Eng.

[CR214] Ziyadanogullari NB (2018). Thermal performance enhancement of flat-plate solar collectors by means of three different nanofluids. Thermal Sci Eng Prog.

